# An investigation into the applicability of the semiempirical method PM7 for modeling the catalytic mechanism in the enzyme chymotrypsin

**DOI:** 10.1007/s00894-017-3326-8

**Published:** 2017-04-04

**Authors:** James J. P. Stewart

**Affiliations:** Stewart Computational Chemistry, 15210 Paddington Circle, Colorado Springs, CO 80921 USA

**Keywords:** Enzymes, Transition states, Catalytic cycle, Intrinsic reaction coordinate (IRC), Protease, Buried aspartate, Oxyanion hole, Catalytic triad, α-chymotrypsin, PM7, MOPAC, Met192

## Abstract

The catalytic cycle for the serine protease α-chymotrypsin was investigated in an attempt to determine the suitability of using the semiempirical method PM7 in the program MOPAC for investigating enzyme-catalyzed reactions. All six classical intermediates were modeled using standard methods, and were characterized as stable minima on the potential energy surface. Using a modified saddle point optimization method, five transition states were located and verified both by vibrational and by intrinsic reaction coordinate analysis. Some individual features, such as the hydrogen bonds in the oxyanion hole, the nature of various electrostatic interactions, and the role of Met192, were examined. This involved designing and running computational experiments to model mutations that would allow features of interest, in particular the energies involved, to be isolated. Three features within the enzyme were examined in detail: the reaction site itself, where covalent bonds were made and broken, the electrostatic effects of the buried aspartate anion, a passive but essential component of the catalytic triad, and the oxyanion hole, where hydrogen bonds help stabilize charged intermediates. With one minor exception, all phenomena investigated agreed with previously-reported descriptions. This result, along with the fact that all the techniques used were relatively straightforward, leads to the recommendation that PM7 and related methods, such as PM6-D3H4, are appropriate for modeling similar enzyme-catalyzed reactions.

**Graphical abstract:**
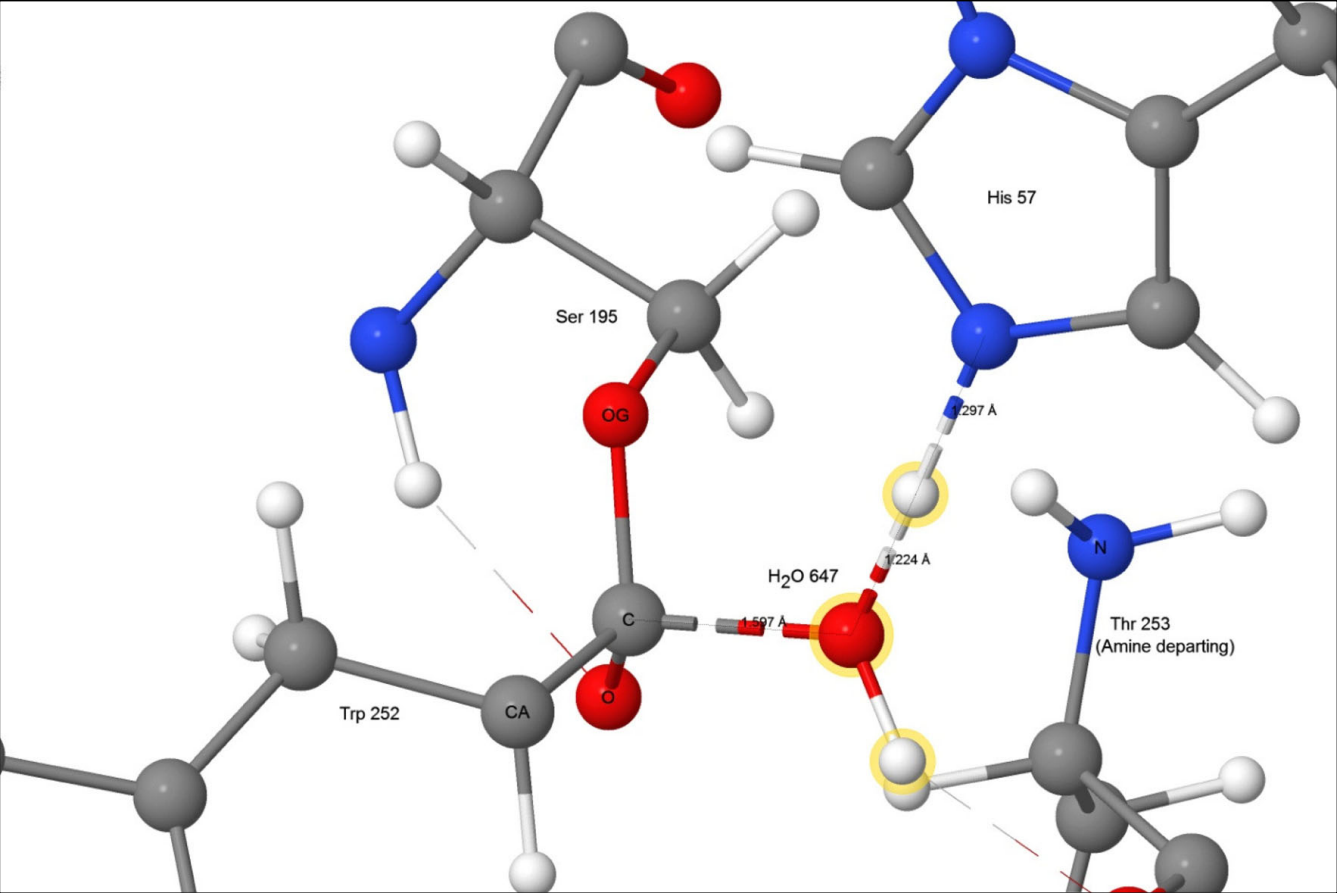
Fifth of six transition states, showing water splitting into hydroxyl anion and a proton, to form the second tetrahedral intermediate and histidinium ion. Atoms of the water molecule involved in the hydrolysis are indicated by halos.

Fifth of six transition states, showing water splitting into hydroxyl anion and a proton, to form the second tetrahedral intermediate and histidinium ion. Atoms of the water molecule involved in the hydrolysis are indicated by halos.

## Introduction

Using computational chemistry methods for mapping out the mechanism of enzyme-catalyzed reactions is both important and difficult. Important, in that an understanding of these mechanisms would be helpful in understanding biochemical processes, and difficult for several reasons: of their nature, enzymes are large biochemicals, methods for modeling chemical reactions necessarily involve solving complicated quantum chemical systems; and, because of the small differences in energies that are involved, a high accuracy is essential if the predictions are to be relied upon.

In general, in order for a mechanism to be mapped using computational methods, two very different types of problems must be addressed. A candidate mechanism for the reaction would first need to be developed, which involves identifying the structures of all the stable intermediates and transition states involved in the catalytic cycle. Collectively, these stable intermediates and transition states are referred to as stationary points on the potential energy surface (PES), a stationary point being characterized by the absence of forces acting on the atoms in the system. The second, more difficult, step, involves modeling these stationary points, i.e., optimizing the geometries of the various systems and then evaluating their energies.

The accuracy of semiempirical methods to reproduce geometries of small systems has steadily increased over the decades. At the same time, the accuracy of the prediction of energies, in particular heats of formation, has also increased, albeit to a lesser extent, so that now the average unsigned error in the most recent PMn method, PM7 [1], for the heat of formation (ΔH_f_) of simple organic compounds is 4.0 kcal mol^−1^. Until recently, the accuracy of prediction of weak interactions, such as hydrogen bonds and van der Waals’ interactions, was low, but in the past few years important advances [2–9] have been made in modeling these weak interactions, which has resulted in a large increase in their accuracy.

Current methods such as PM7 are computationally rapid enough for entire protein geometries to be modeled using readily-available computers. This has resulted in semiempirical methods being used to detect and correct [10] errors in PDB [11] structures, and to improve protein structures [12] from a chemical perspective by making small changes to atomic positions in PDB geometries. Semiempirical methods have also been shown to be useful [13, 14] in modeling various structures in the region of a substrate—enzyme environment.

For convenience, all calculations were performed using PM7, but similar results would be expected if any of the closely-related improved variants of PM6 [15], in particular PM6-D3H4 [9], were used.

In this investigation, the suitability of PM7 for modeling a complete catalytic cycle of the protease α-chymotrypsin was investigated. Chymotrypsin is a protease that selectively hydrolyzes peptide bonds between aromatic residues [16, 17]. Because it has been well-studied [18] it provides an ideal system for evaluating the applicability of PM7 to enzyme catalyzed reactions. In addition, the presence of two specific, very interesting, and highly compact features within chymotrypsin—the catalytic triad and the oxyanion hole—play an important role in the catalytic process. The ability of these structures to influence the activation barriers is therefore of great interest.

The investigation involved three stages. In the first stage, all stable intermediates were identified and refined using well-established techniques. In the second stage, all transition states for reaction steps where intermediates were separated by a reaction barrier were modeled. A new transition location method, LOCATE-TS, tailored for use with biochemical reactions, was developed to simplify this task.

The third stage involved predicting the energies of various interactions. Unlike the other two stages, no equivalent currently exists in experimental chemistry for the quantities being investigated. Instead, these quantities represent abstract, but nonetheless very useful, theoretical concepts. For example, at one point in the catalytic process a charge separation occurs when a proton migrates from one residue to another. This results in the generation of various electrostatic interactions that are crucial to the catalytic process, but since a large number of other phenomena occur simultaneously, isolating and evaluating the energy of any specific electrostatic interaction would be difficult. However, by designing computational experiments that model mutations, each specific interaction of interest can be isolated and its energy calculated. This technique could then be applied to other phenomena, such as predicting the stabilization energy of the hydrogen bonds in the oxyanion hole in the second catalytic step.

During the simulations, a residue that was not in the active site, Met192, was predicted to move significantly. An investigation of its motion indicated that Met192 contributed to the catalytic activity.

## Computational details

### Geometry optimization

Two different procedures were used in preparing optimized geometries. The first procedure was used only once in the preparation of the starting model, a geometry that would be used as the starting point for all points in the catalytic cycle. The objective was to generate a system that had an energy that was an irreducible minimum. This could be defined as a geometry that, if it were subjected to a small conformational distortion, a normal geometry optimization operation would restore it to its undistorted state and not to some alternative conformation that had a lower heat of formation. The other procedure was used to optimize the geometry of all points that were modeled. Both procedures used the COSMO [19] solvation model, and geometry optimization was performed using the efficient [20] L-BFGS method [21, 22].

The first procedure mimicked simulated annealing. After a normal geometry optimization was performed, minor conformational changes were made to various sites in attempts to make a more stable structure. Normal geometry optimization was then carried out after each modification. From the resulting set of structures the system with the lowest energy was selected as being the most stable.

In the second procedure, the geometry of each system selected was re-optimized, and the optimization continued until the heat of formation failed to descend over a further 100 cycles. This exhaustive optimization increased the likelihood that the system was at an irreducible minimum. Note that, because large conformational changes were not allowed, this minimum might not coincide with the global minimum.

### Locating transition states

Several very different methods have been developed for locating transition states. Among the more popular are methods based on the use of synchronous transit interpolation [23]. Using linear-scaling density functional theory together with the synchronous transit method, Lever et al. [24] located the transition state geometry for the enzyme-catalyzed rearrangement of chorismate to prephenate, demonstrating that ab initio methods could be used for modeling individual steps in biochemical reactions.

An alternative approach uses structures derived from the reactants and product geometries, together with a bias potential, to locate the region of a transition state. This double-ended surface walking (DESW) method was demonstrated [25] to be more efficient than earlier methods, and was successfully used [26] in the generation of the intrinsic reaction coordinate for the first step in the synthesis of isopenicillin.

An earlier form of the DESW method was used for modeling the first step in the chymotrypsin mechanism [27], but as with many transition state location methods, this method had a specific limitation. Capabilities of a new transition location method are often demonstrated by its application to one or more sample reactions: these demonstrations require a significant amount of specialist skill if they are to be successful. This presents an issue for the intended end users, in particular experimentalists who do not possess computational modeling expertise, and problems frequently occur that make success elusive. Therefore, an additional objective of the current work has been to develop a method that simplifies the technical aspects of locating transition states in biochemical processes, and to eliminate as many as possible of the problems that experimental, i.e., non-computational, chemists encounter. If this objective were to be achieved, the range of applicability of semiempirical methods would be enhanced by shifting the focus of the work from the technical minutia of the computational process to the considerably more important investigation of the machinery of the catalytic system involved.

#### Methodology

**Fig. 1:**
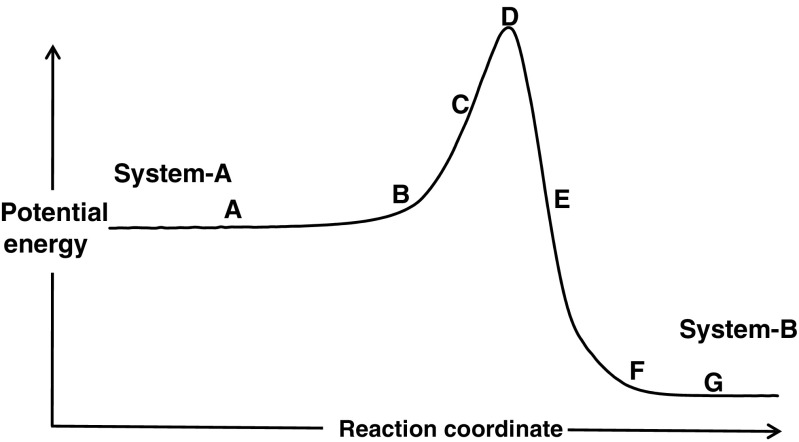
General potential energy diagram for a chemical reaction

Individual chemical reactions in an enzyme-catalyzed process can be represented by a potential energy diagram showing motion along a hypothetical reaction coordinate from reactants to products, as shown in Fig. 1, which illustrates the various features of a generic reaction that will be discussed later on. For convenience, system-A can be regarded as the reactant, and system-B as the product, but as individual reactions in enzyme-catalyzed processes are reversible, the terms “reactant” and “product” should not be interpreted literally.

In any given reaction the reactants, transition state, and products can be represented by stationary points on the PES. In the case of reactants and products, these stationary points are energy minima and are relatively easy to model using routine geometry optimization methods. Unfortunately, automating the optimization of the geometry of a transition state still “remains a challenge [28],” given that the stationary point is the highest point on the lowest-energy path connecting reactants and products. Expressed more formally, a transition state is a stationary point on the PES that has precisely one negative force constant, and the associated eigenvector (normal mode of vibration or normal coordinate) points in the direction of the reactants and products.

Procedures for locating a transition state typically begin with two known stationary points separated by an energy barrier that are used as the starting points for locating the approximate position of the transition state. If the resulting approximate position is near enough to the exact transition state, then conventional gradient minimization methods can be used to refine the transition state stationary point.

The procedure used here follows the same general approach. Two geometries, one, *X*_***A***_, representing the reactant system, **A**, the other, *X*_***G***_, representing the product system, **G**, in Fig. 1, are superimposed and the difference between them, *R*_***AG***_, evaluated:

$$ {R}_{\boldsymbol{A}\boldsymbol{G}}=\sqrt{{\displaystyle \sum_i}{\left({X}_{\boldsymbol{A}}^i-{X}_{\boldsymbol{G}}^i\right)}^2}\kern0.5em . $$

By definition, because **A** and **G** were generated by unconstrained global optimizations, their heats of formation would both be minima on the PES. If the unconstrained global optimization of **A** or **B** were to be replaced with a constrained global optimization in which a bias was added to the heat of formation calculation, a bias that increased in proportion to the square of the distance between the two stationary points, i.e., *R* ^2^_***AG***_, then geometry optimization of **A** while geometry **G** was held constant would result in a structure displaced toward **G**, and vice versa. That is, by adding an energy bias to the geometry optimization procedure, the geometries of **A** and **B** could be moved nearer to that of the transition state.

In this work, the use of a bias toward a fixed structure was avoided by replacing the two separate constrained global geometry optimizations with a single optimization that minimized the sum of the two heats of formation simultaneously. To achieve this, the individual heat of formation calculations were replaced with the combined function shown in Eq. 1, where *c* is a scalar constant representing the bias, and **A′** and **G′** represent the two systems as the distance between them decreases.

1 $$ \varDelta {H}_f^{\prime }=\varDelta {H}_{f A\prime }+\varDelta {H}_{f G\prime }+ c{R^2}_{A\prime G\prime } $$

Each evaluation of the function being minimized, Δ*H′*_*f*_, thus involved three separate calculations, for Δ*H*_*f****A′***_, Δ*H*_*f****G′***_, and *R* ^2^_***A′G′***_.

### General technique for locating, refining, and validating a transition state

All transition states were located, refined, and validated using the same multi-step technique. As with all transition state location methods, this technique should not be regarded as being fully automatic in that each transition state in the system being modeled had to be examined carefully, including inspection using a graphical user interface, to verify its validity. Almost all of the problems encountered involved erroneous assumptions being made regarding the catalytic process, for example, using the wrong ionization state of a carboxylate group. Most of these would be revealed when a visual examination of the system was carried out.

#### Initial motion to the start of the reaction barrier

Fully optimized protein geometries of the type represented by the stationary points **A** and **G** in Fig. 1 are extremely flexible, and even quite large displacements would incur only a small energy penalty. This is represented by the extended, essentially constant, potential energy in the regions of points **A** and **G**. In these domains, motion in the direction of the transition state would not result in any significant increase in energy until the bottom of the potential energy barrier was reached, at points **B** and **F**. The first step in locating the transition state, then, would be to optimize the geometries using a small bias: in practice, a suitable initial value would be in the range *c* = 1 to 3 kcal mol^−1^ Å^−2^.

During this geometry optimization a significant energy penalty would only be incurred by those atoms directly involved in the reaction as they moved slightly up the barrier of the transition state; motion of all the other atoms would involve a negligible energy penalty. The result of this operation would be to reduce *R* ^2^_*A′G′*_ from possibly several hundred Ångstroms to less than 40 Å.

#### Motion up the barrier to the inflection point

A stepwise increase in the bias *c* results in motion up the barrier. For any given value of *c*, the resulting optimized geometry would represent the equilibrium between the force exerted by the bias and the gradient due to distortion from the optimized geometry. At the equilibrium geometry the forces on **A′** and **G′** would be equal in magnitude but opposite in direction, and the positions of both systems on the reaction coordinate would be such that the slopes would also be equal in magnitude and opposite in direction. Obviously, this does not imply that the heats of formation of **A′** and **G′** would be the same or that the distance from the transition state to **A′** and **G′** would be the same.

#### At the inflection point

With increasing bias the optimized geometries would move steadily up the barrier, eventually passing the inflection points, **C** and **E**, that is, the points where the slope no longer increases. In principle, in the region above both inflection points, minimization of the gradient should result in optimization to the transition state stationary point, but in practice this normally does not occur. When attempts to refine the transition state were made using commonly-available gradient minimization methods such as Baker’s Eigenfollowing [29] method, the system either optimized to an energy minimum or the optimization procedure ended when an error was detected.

#### Above the inflection point

Continued motion in the direction of the transition state could be achieved by steadily increasing the bias. If this is done, then the slope of the PES would steadily decrease, resulting in the effect of the force from the bias becoming more important, but, at the same time, the decreasing distance across the barrier would result in a reduction in the magnitude of that force. Together, the combination of these effects results in the system continuing to move toward the transition state as the bias is increased. Only in the immediate vicinity of the transition state, point **D**, a zone in which the barrier profile becomes increasingly parabolic, does the effect of increasing bias become deleterious, with the commonest drawback being that one of the systems moves through the barrier to the other side; once this occurs, any subsequent geometry optimization would result in a catastrophic descent to either the starting reactant or product geometry, depending on which system crossed the barrier.

This undesirable result can be easily avoided by monitoring the angle between the gradient vectors for the two systems. While both geometries are on different sides of the transition state, the angle between these two vectors would be near 180°. Any significant decrease in angle would indicate the potential onset of instability, at which point the ascent of the barrier could be terminated. If the angle between the gradient vectors remained near 180°, then the ascent of the barrier could be continued until *R*_***A****'****G****'*_ became sufficiently small, typically less than 3.0 Å, so that, if a transition state refinement were to be performed, there would be a high probability of its success.

A good approximation to the transition state geometry would be obtained by averaging the final two geometries, regardless of whether they were obtained by terminating the ascent due to incipient instability or by the distance on the PES between the systems dropping below a pre-set limit.

#### Refining the transition state

The geometry resulting from the ascent of the potential energy barrier would be near to, but not at, the transition state, in that there would still be small forces acting on the atoms. Refinement of the geometry involves moving it to the stationary point. In principle, this could be achieved by a global gradient minimization, but such an operation would be computationally very demanding. An alternative, much less demanding, approach involves partitioning the atoms into two sets. One set consists of those atoms that are involved in bond making and bond breaking in the chemical reaction and, optionally, some nearby atoms. The other set consists of all the other atoms. Refinement of the transition state could then be achieved by performing a gradient minimization on the first set while holding the atoms of the second set fixed, followed by a geometry optimization, i.e., energy minimization, of the second set while holding the atoms of the first set fixed. Each of these operations would result in changes in the forces acting on the fixed atoms in the other set. These induced forces could then be easily eliminated by repeating the operation and, since this is a rapidly-converging series, this could be achieved in two to five iterations.

Identifying the atoms involved in bond making and bond breaking is straightforward. The topologies of the two starting systems would be compared, and, when a difference in the connectivity of an atom is detected, then that atom must be involved in bond fission or fusion and would therefore be assigned to the first set; normally this consists of 2 to 4 atoms. If nearby atoms are included, then all atoms connected to any atoms in the first set would also be added to that set, but because the increase in the number of atoms used in the gradient minimization would result in a significant increase in the computational effort, the use of this option should be avoided when possible.

#### Heat of formation for transition states

All work involving comparisons of heats of formation require high precision. In the case of stable intermediates, this precision could be achieved by using the second geometry optimization procedure described earlier. By making a slight modification to that procedure, geometries of transition states could also be optimized to the necessary high precision.

Geometries resulting from transition state refinement could be regarded as being composed of two sets: those atoms directly involved in the reaction, and all other atoms. As a result of this refinement, the positions of all atoms in the first set, typically seven or fewer, would be highly optimized. By freezing the positions of these atoms and then running the second geometry optimization procedure, the geometry of the entire transition-state system could be optimized to the same precision as that used for stable intermediates. In test calculations, a gradient minimization was performed using the atoms in the first set to verify that any spurious forces introduced by this geometry optimization did not cause significant changes. In all cases, this resulted in changes of less than 0.2 kcal mol^−1^ in the heat of formation, and less than 0.001 Å in the positions of the atoms.

Performing an exhaustive geometry optimization on the transition state geometries ensured that the resulting heats of formation were directly comparable with those of the various intermediates.

#### Reducing computational effort

Each complete step in moving up the barrier involves a minimization of the combined energy of both systems and the energy arising from the bias. In conventional geometry optimizations, every point calculated normally involves a complete self - consistent field (SCF) calculation. However, with the exception of the first step, all the steps in locating the transition state involve only small movements of the atoms, and therefore only minor perturbations to the wavefunction. This opens the possibility of eliminating some of the SCF calculations and thus reducing the computational effort required. Timing tests led to the conclusion that the most efficient option was to perform a single SCF calculation at the start of each step, and to use the results of that initial SCF calculation in all subsequent geometric operations within that step. When this modification was made, the complete operation of locating and refining a transition state, given initial reactants and products, required roughly the same computational effort as a normal unconstrained geometry optimization of a system of the same size.

For all systems modeled, that is for all reactants and all products of a reaction, the sequence of events that occurred during the ascent of the reaction barrier was the same. At the bottom of the barrier the initial rate of ascent was small but increased steadily until the inflection point was reached, then decreased again as the transition state was approached. At that point the shape of the PES became a simple parabola, implying that the shapes of the PES in the forward and reverse directions were identical. That is, in the region of the transition state, all information regarding those properties that had been specific to the reactants and products, such as bonding, geometry, and heat of formation, was lost. This high symmetry only exists in the immediate vicinity of the transition state: as the barrier was descended, the system would once again take on the character of the reactant or product system.

### Validation of transition states

Transition states for a reaction are characterized by the presence of a single normal mode of vibration connecting the two systems involved in that reaction. Normal modes representing motion through the transition state are identified by a large imaginary vibrational frequency; all other normal modes have a real, positive frequency. The fact that reaction coordinate modes must necessarily be well-separated from all other modes implies that they are highly localized, i.e., that they would have significant intensity on only those atoms that were involved in the reaction. This means that only those atoms that are directly involved in the reaction [27] are needed in the vibrational analysis in order to verify that the system is, in fact, a transition state. When this is done, the computational effort decreases significantly and all irrelevant imaginary vibrations, such as rotation of methyl groups, are automatically eliminated. If the only atoms used in the vibrational analysis were those involved in the making and breaking of covalent bonds, then, although the presence of a single large imaginary mode would be indicative of a valid transition state, its value would be perturbed because the normal coordinate would also have some intensity on nearby atoms. If desired, this perturbation could be reduced by the simple expedient of including the nearby atoms in the vibrational frequency calculation.

### Intrinsic reaction coordinate

In a chemical reaction the intrinsic reaction coordinate (IRC) [30] is the combination of the two mass-weighted steepest-descent paths on the PES from the transition state to the energy minima. The transition state geometry itself, being a stationary point on the PES, is unsuitable as a starting point for this path, but if the geometry were to be perturbated by a small displacement in the direction of the transition state normal mode, then the system would no longer be at a stationary point but instead would be on the downward slope. Once on that slope, the rest of the path could be generated using the forces acting on the atoms.

The other half of the reaction coordinate could then be generated in a similar manner by reversing the direction of the displacement.

### Derived properties

Some derived quantities are useful in describing individual features of interest in the catalytic process. None of them are directly observable by experiment, and so the definition of each derived quantity cannot be related to any definable physical quantity but, by conducting computational simulations of hypothetical reactions, estimates of their values can be obtained. For example, given that the catalytic influence of the Asp102 anion had to be a result of the electrostatic effects of its negative charge, deleting that charge would be expected to remove its influence and therefore an examination of the changes resulting from neutralizing the charge should reveal useful information about its role. The simplest model for the neutralization of the charge on residue 102 would be to replace its carboxylate group by a hydrogen atom to form the mutant D102A.

#### Electrostatic phenomena

Electrostatic interactions between various charged entities in the region of the reaction site are essential to the catalytic process. An estimate of the value of these interactions was obtained using the following generic procedure.

Given two charged sites, A(±) and B(±), where " ± " represents the presence of either a positive or a negative charge, electrostatic theory indicates that the interaction of these charges would result in an energy, either positive, if the charges had the same sign, or negative, if the signs were different. At different points in the catalytic cycle various charges exist in the unmodified system. For example, in all intermediates, Asp102 had a net negative charge, while, in those intermediates where a proton was being shuttled, His57 had a positive charge.

When one of the charges is neutralized, i.e., A(0) or B(0), the corresponding electrostatic energy between A and B would vanish. This allows the electrostatic interaction energy to be calculated.

Consider the four systems:

**Table:** 

System	Components	Description	Electrostatic interaction
A	A(±)B(±)	Both sites are ionized	Present
B	A(0)B(±)	Site "A" neutralized	Absent
C	A(±)B(0)	Site "B" neutralized	Absent
D	A(0)B(0)	Both sites neutralized	Absent

The electrostatic interaction, E_el_, could then be calculated from the heats of formation of the four systems using Eq. 2.

2 $$ {\mathrm{E}}_{\mathrm{el}}=\Delta {\mathrm{H}}_{\mathrm{f}}\left(\mathrm{A}\right) + \Delta {\mathrm{H}}_{\mathrm{f}}\left(\mathrm{D}\right)\ \hbox{-}\ \left(\Delta {\mathrm{H}}_{\mathrm{f}}\left(\mathrm{B}\right) + \Delta {\mathrm{H}}_{\mathrm{f}}\left(\mathrm{C}\right)\right) $$

Although Eq. 2 is simple, in order for the resulting electrostatic interaction to be meaningful, two limitations or caveats must be applied:

1. As with all quantum chemical simulations, each neutralized system must be chemically sensible. In some cases, such as Asp102(−), a simple mutation to replace the ionized group by a hydrogen atom, as in the mutation D102A, or to remove a proton, e.g., His57(+) going to His57(0), would be sufficient. In others, replacing an atom with another atom that is one higher or one lower in the periodic table can achieve the desired neutralization. For example, in step 2 (Fig. 2), the formal charge of −1 on the peptide oxygen atom in Trp252 could be neutralized by replacing the oxygen by a fluorine atom.

**Fig. 2:**
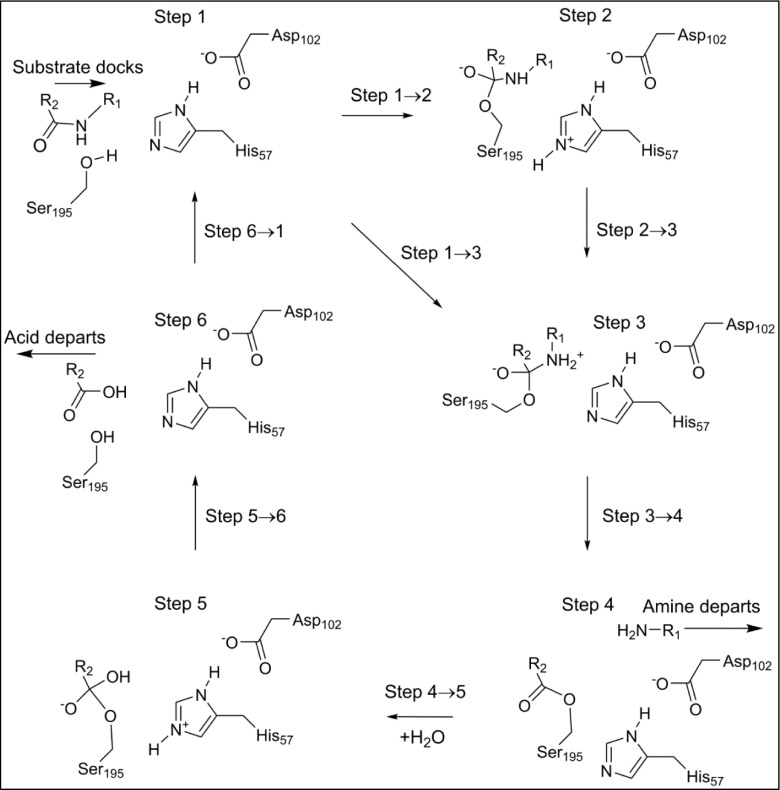
Stable intermediates in the chymotrypsin catalytic cycle. -R_1_: −Thr_253_-NH_2_ -R_2_: −Trp_252_-Ala_251_-Gly_250_
2. When possible, in order to minimize various errors, the geometry of most of the system being calculated should be held frozen, the exception being the site of the mutation itself. Thus, in the D102A mutation, the positions of the methyl hydrogen atoms would need to be optimized, as would the position of the fluorine atom in the O(−) → F(0) mutation.

#### Hydrogen bond energies

Although no direct prediction can be made of the energies of individual hydrogen bonds, if the strength of a hydrogen bond changes during a reaction then an estimate can be made of the corresponding change in energy. The technique used for predicting changes in hydrogen bond energies involves a simple modification of the technique used in estimating electrostatic interaction energies.

Hypothetically, hydrogen bond energies could be predicted by calculating the energy of a system with the hydrogen bond present and then with the hydrogen bond absent. Individual hydrogen bonds could be deleted by eliminating the appropriate hydrogen atom. To prevent this operation from resulting in an unrealistic chemical system, the atom that the hydrogen was attached to would need to be replaced by an atom of the next higher atomic number. In practice, this means that a nitrogen donor atom would be replaced by an oxygen atom, and an oxygen donor atom would be replaced by a fluorine atom.

Calculation of the change in hydrogen-bond energy for a pair of points, A and B, in the catalytic mechanism requires modeling four systems. In order, these are:

**Table:** 

System	Description	Hydrogen bond
H(A)	System A, unmodified	Present
H(B)	System B, unmodified	Present
X(A)	System A, mutated	Absent
X(B)	System B, mutated	Absent

If a hydrogen bond of interest were to be deleted by replacing the donor system, O-H or N-H, by a single atom, then any other hydrogen bonds to that donor would also be deleted. Eliminating the resulting geometric strain energy almost always necessitates a global re-optimization. This can be contrasted with the situation in the electrostatic energy calculation where the positions of only a few atoms in each mutant system had to be optimized.

Using these systems, the change in hydrogen bond energy, ΔE_Hb_, in going from system A to system B is given by Eq. 3.

3 $$ \Delta {\mathrm{E}}_{\mathrm{H}\mathrm{b}}=\Delta {\mathrm{H}}_{\mathrm{f}}\left(\mathrm{H}\left(\mathrm{B}\right)\right)\ \hbox{-} \Delta {\mathrm{H}}_{\mathrm{f}}\left(\mathrm{H}\left(\mathrm{A}\right)\right)\ \hbox{-}\ \left(\Delta {\mathrm{H}}_{\mathrm{f}}\left(\mathrm{X}\left(\mathrm{B}\right)\right)\ \hbox{-} \Delta {\mathrm{H}}_{\mathrm{f}}\left(\mathrm{X}\left(\mathrm{A}\right)\right)\right) $$

## α-chymotrypsin

Chymotrypsin consists of three polypeptide chains held together by disulfide bonds. It is generated by the selective hydrolysis of various peptide bonds in its precursor chymotrypsinogen, a single chain protein consisting of 245 residues. Of the large number of entries for chymotrypsin in the PDB, 8GCH was selected as being the most suitable for the construction of a starting system for modeling the catalytic cycle because of the presence of a specific tripeptide fragment docked in the active site. This substrate, Gly-Ala-Trp, was a product of autolysis, and its position and orientation provided an excellent starting point for the construction of a hydrolysable substrate. The sequence Gly-Ala-Trp occurs in chymotrypsin precisely once, in residues 205–207, and since the Trp carboxylate terminus is located in close proximity to the residue Ser195, the inference was made that hydrolysis had occurred at the peptide bond between Trp207 and the next residue in chymotrypsin, Thr208: that is, at the peptide bond Trp207-Thr208. The substrate had therefore consisted of the sequence Gly-Ala-Trp-Thr plus an additional polyatomic group of unknown size. Although the next residue was Leu, there was no compelling reason to increase the size of the substrate, so for convenience Leu was replaced by the simple polyatomic group -NH_2_.

Preparation of a starting model began by adding Thr-NH_2_ to the tripeptide substrate. In 8GCH, the tripeptide substrate had been given residue numbers 250-252, so, for consistency, the residue numbers for the substrate to be used in the starting model were assigned as Gly250-Ala251-Trp252-Thr253-NH_2_.

The starting model consisted of the three polypeptide chains (chain E, consisting of residues Cys1-Ser11, chain F, consisting of Ile16-Tyr146, and chain G, consisting of Asn150-Asn245), the substrate (chain C, consisting of Gly250-Thr253), four sulfuric acid molecules, and 347 water molecules. Earlier work had shown [14] that, unless a potentially ionized site was near the site of interest (here the active site) its state of ionization was unimportant. For this reason, after the system was hydrogenated the sulfate groups reported in the PDB were left unionized. One residue near the active site, Asp102, is generally accepted as being ionized, so its carboxylic acid group was deprotonated. Hydrogenation and ionization of Asp102 resulted in the system having the empirical formula C_1115_H_2449_N_302_O_704_S_16_, for a total of 4,586 atoms, and a net charge of −1.

Preparation of the starting model was completed by performing the two geometry optimization procedures described earlier.

The starting model corresponded to step 1 of the catalytic mechanism used by chymotrypsin as shown in Fig. 2. Three salt bridges formed during the construction of the starting model, between Ser11 and Glu20, Arg154 and Glu21, and Ile16 and Asp194. The first two of these salt bridges were on the surface of the protein, far from the active site, and therefore would have no significant effect on the catalytic cycle. The third salt bridge, between Ile16 and Asp194, was essential for the catalytic behavior in that it helped shape the geometry of the oxyanion hole that, in turn, helped to stabilize two of the intermediates.

A comparison of the geometry of the active site in 8GCH and the equivalent optimized PM7 geometry gave a RMSD of 0.388 Å, with the largest difference in any atom’s position occurring in the peptide oxygen of Trp252. This particular atom would be expected to be significantly affected because of the large change resulting from the reconstruction of the substrate.

In order to permit a valid comparison to be made of the various stages of the catalytic cycle, every model used had to have exactly the same general composition, the same empirical formula, and the same charge. Near the end of the mechanism an ester group is hydrolyzed, and in that process a water molecule is decomposed. To accommodate this, a water molecule in 8GCH, that had originally been interacting with the ionized carboxylate group of Trp252, H_2_O-647, was re-positioned to be nearer to where the ester group would eventually be located.

Because standard heats of formation for all systems investigated were very large and negative, for convenience all heats of formation reported here are relative to the heat of formation of step 1, ΔH_f_ = -47899.77 kcal mol^−1^.

### Catalytic cycle

Six stable intermediates, step 1 through step 6, are involved in the overall reaction (Fig. 2), each represents a local minimum on the PES. Progression through the catalytic cycle is indicated by the various steps representing motion from one intermediate to the next. Steps 1 → 2, 2 → 3, 1 → 3, 4 → 5, and 5 → 6 represent classical reactions, with activation barriers and transition states. In step 3 → 4, two essentially activationless reactions occur: the amine generated by hydrolysis of the peptide bond migrates out of the active site and a water molecule migrates into the active site. In step 6 → 1, a similar activationless reaction occurs when the other product of hydrolysis, the tripeptide, migrates out of the active site and a new substrate enters the active site.

### Use of the potential energy surface

For computational convenience all models were represented using the potential energy surface concept, in which the energy and geometry of a chemical system are represented by a point on a multi-dimensional surface. Each dimension corresponds to one degree of geometric freedom; in the system being modeled, with 4,586 atoms, this amounted to 13,758 dimensions. The PES was represented in Cartesian coordinates, which are continuous and single-valued; internal coordinates were unsuitable because they are discontinuous and because individual atom coordinates and gradients would depend on the atoms’ connectivity. Mass-weighting was not used when distances were calculated, but was used when IRC paths were generated.

All stationary points, i.e., all ground and transition states, were represented as individual points on this hypersurface. In addition, simple geometric quantities can be defined, among the more important being the distance between individual stationary points and the angle between any transition state and its corresponding reactants and products.

Semiempirical methods are parameterized to reproduce chemical properties at the standard state temperature of 298.15 K, so the semiempirical PES, instead of representing the formal Born-Oppenheimer surface, i.e., the surface at 0 K, should be regarded as representing the surface at the standard-state temperature.

## Results

### Individual intermediates

In preparation for a detailed examination of the various reactions in the catalytic cycle an unconstrained global optimization was performed on each of the six intermediates shown in Fig. 2. These stationary points on the PES were then used by the procedure described earlier for locating and refining the transition states.

#### Step 1

**Fig. 3:**
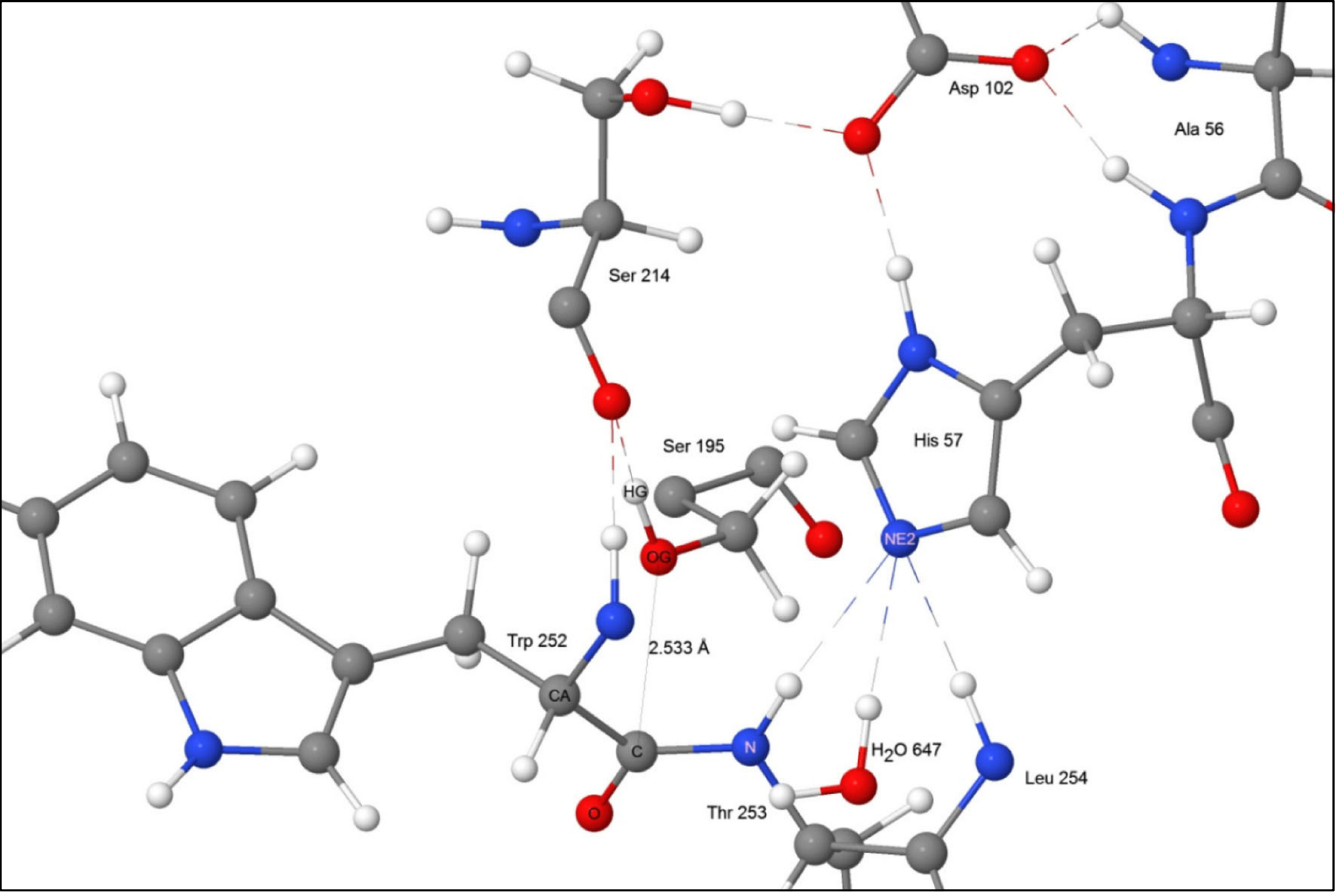
Step 1: starting geometry showing residues Trp252 and Thr253 of the substrate docked into the binding site

At the start of the reaction the substrate was docked in the active site and held in place by a set of hydrogen bonds and van der Waals’ interactions. Near to it was the catalytic triad composed of the three residues His57, Asp102, and Ser195. One of these residues, the ionized Asp102(−), formed four hydrogen bonds with nearby residues, with the most important being between O_δ2_ and the H_δ1_ on the imidazole group of His57. During the course of the reaction cycle Asp102(−) did not take an active part in any of the steps. The N_ε2_ of His57 formed a normal hydrogen bond with the hydrogen atom on the peptide nitrogen of Thr253 as shown in Fig. 3.

In the optimized PM7 geometry, the hydroxyl hydrogen on residue Ser195 formed a normal hydrogen bond with the peptide oxygen atom of Ser214. This hydrogen bond orientated the hydroxyl oxygen atom so that it was only 2.53 Å from the peptide carbon of Trp252. That is, the hydroxyl oxygen of Ser195 was ideally oriented for reacting with the peptide bond that would be hydrolyzed.

#### Step 2

**Fig. 4:**
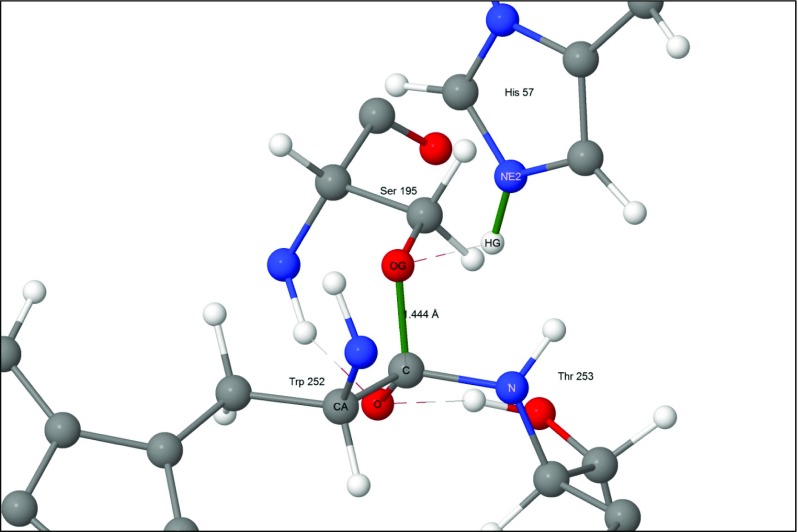
Step 2: the first tetrahedral intermediate showing the newly-formed covalent bonds in green

**Fig. 5:**
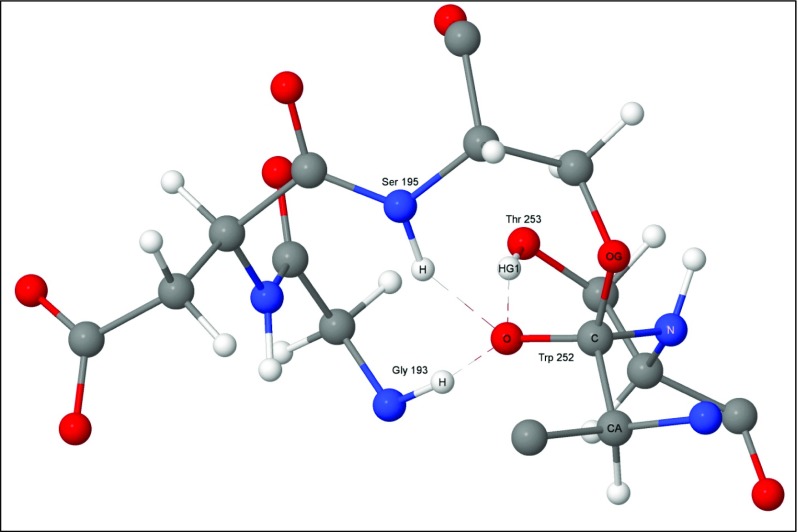
Step 2: the first tetrahedral intermediate and the oxyanion hole

Two new bonds were formed by the first reaction, with the more important one, Fig. 4, generating a Michaelis complex by connecting the peptide carbon of substrate residue Trp252 to the hydroxyl oxygen, O_γ_, on enzyme residue Ser195. As indicated in Fig. 2, the presence of this bond implies that the peptide oxygen on Trp252 would have a significant negative charge. This negative charge would then be stabilized by the hydrogen bonds in the oxyanion hole, Fig. 5. For convenience, the term "oxyanion hole" will be reserved for the three residue structure Gly193-Asp194-Ser195, and the "oxyanion site" for the same structure plus the substrate residues Trp252 and Thr253. In the system being investigated the oxyanion site contains three hydrogen bonds to the anionic oxygen of Trp252, one to each of the amide nitrogen atoms of Gly193 and Ser195 in the oxyanion hole, and one to the hydroxyl oxygen of the substrate residue Thr253.

#### Step 3

**Fig. 6:**
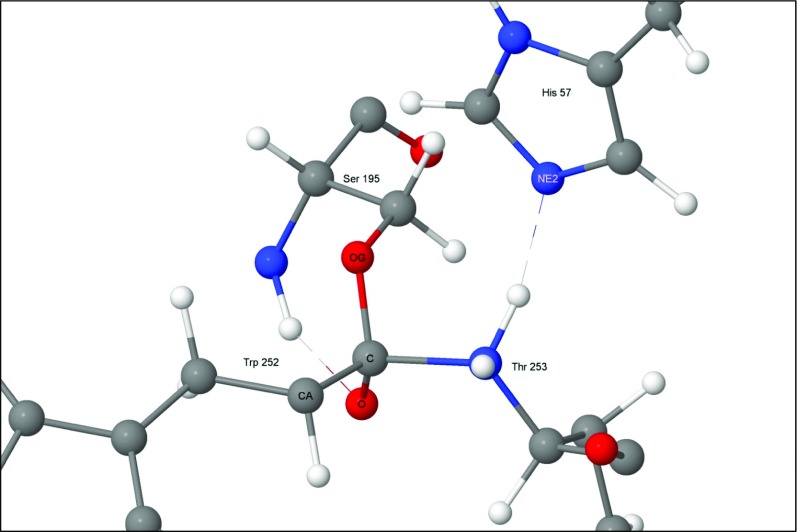
Step 3: hydrogen on peptide nitrogen of threonine 253

Although step 3 is a minimum on the PES, it is highly unusual in that it connects a cationic site, the quaternary nitrogen, to an anionic site, the tetrahedral carbon anionic site (Fig. 6), and, as such, it has some zwitterionic character. If the weak covalent amide bond were to break, then both charged sites would be neutralized, a process that would be essentially activationless and therefore could be presumed to occur spontaneously at in vivo temperatures.

#### Step 4

**Fig. 7:**
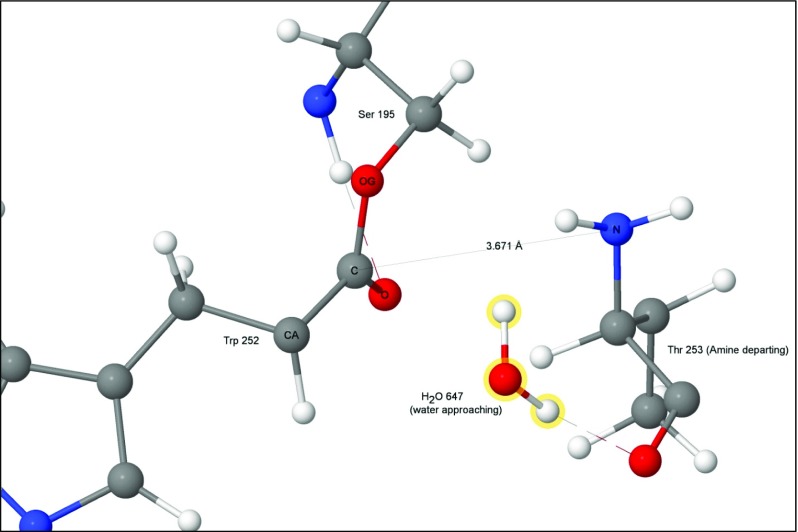
Step 4: threonine 253 migrated away from tryptophan 252, acyl complex left behind. Atoms of the water molecule involved in the hydrolysis are indicated by halos

An acyl-enzyme intermediate forms when the amide C-N bond breaks. The amine resulting from the fission migrates away from the reaction site, and a water molecule, H_2_O-647, diffuses into a position (Fig. 7) where it can react with the ester group.

#### Step 5

**Fig. 8:**
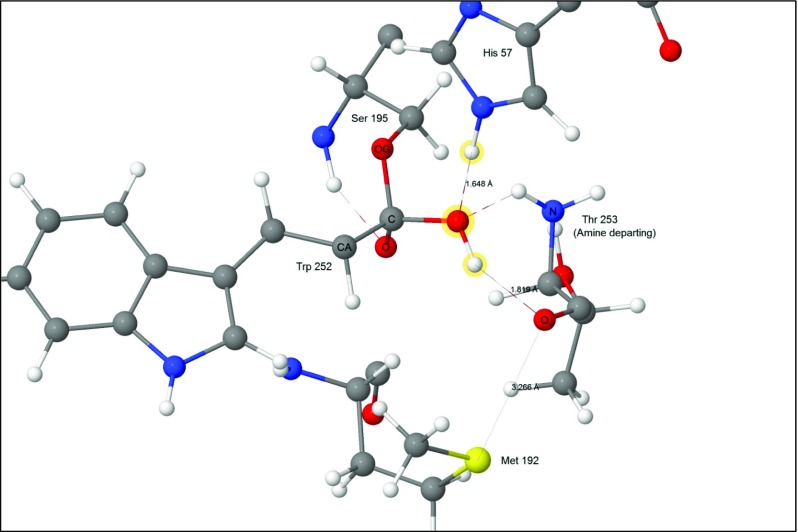
Step 5: water split to form the second tetrahedral intermediate and the second ion-pair Asp102(−) - His57(+). Atoms of the water molecule involved in the hydrolysis are indicated by halos

H_2_O-647 splits to form a proton and a hydroxide anion which covalently bonds to the peptide carbon on Trp252. Part of the charge on the hydroxyl group transfers to the carbon atom to form a second tetrahedral intermediate. At the same time, the proton from H_2_O-647 migrates to N_ε2_ of His57. As with the first tetrahedral intermediate, the charge separation that occurs in this system (Fig. 8) would also be stabilized by the oxyanion hole and by the residues Asp102(−) and His57 of the triad.

An intermediate stable system had been predicted to exist between step 5 and step 6. In step 5 the ionizable proton on His57 forms a strong hydrogen bond with the hydroxyl oxygen on the tetrahedral intermediate. Topf and Richards predicted [31] that, in the reaction step 5 → 6, a barrier would exist between the proton hydrogen-bonding to the hydroxyl oxygen and the proton hydrogen-bonding to O_γ_ of Ser195, and that the proton hydrogen-bonding to O_γ_ of Ser195 would be slightly lower in energy. Attempts to model this reaction were unsuccessful. PM7 predicted that the system in which the proton was hydrogen-bonding to the hydroxyl oxygen had the lower energy. No stationary point was identified in which the proton was hydrogen-bonding to O_γ_ of Ser195; instead, only a metastable structure was found. When this was allowed to relax, the proton migrated to form a bridging hydrogen bond with both the hydroxyl oxygen and the O_γ_ of Ser195. This bridging hydrogen bond then decomposed to give the geometry shown in Fig. 8. At all points in this process the heat of formation decreased monotonically.

#### Step 6

**Fig. 9:**
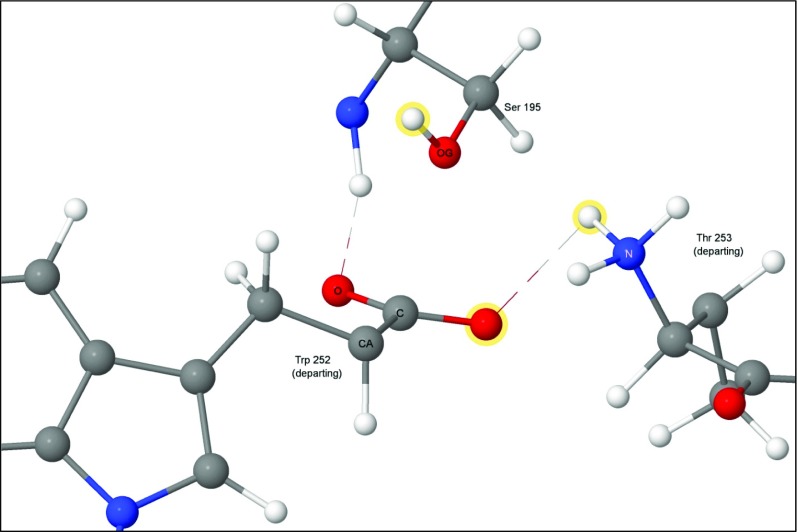
Step 6: reaction complete; products depart. Atoms of the water molecule involved in the hydrolysis are indicated by halos. The proton on Ser195 O_γ_ migrated from His57, and the proton on Thr253 migrated from the hydroxyl hydrogen on Trp252, resulting in the formation of a salt bridge.

At the end of the cycle the Michaelis complex is destroyed when Ser195 is regenerated as a result of the fission of the Ser195 O_γ_ - Trp252 C ester bond. This occurs when the proton that was on His57 migrates to Ser195 O_γ_ (Fig. 9), and the hydroxyl proton migrates to the departing amine, generating a salt bridge.

### Transition states

#### Locating and refining transition states

All individual transition states were located using the technique described earlier. To recap: For each reaction where a barrier separated reactants and products, a calculation was performed in which the distance between the two geometries representing the two stationary points was systematically reduced using Eq. 1. This procedure resulted in a geometry that was near to the transition state. A gradient minimization procedure was then used to refine the transition state. These two procedures were run consecutively within the MOPAC2016 program [32] using the new keyword LOCATE_TS. This was followed by an exhaustive geometry optimization of all atoms that were not directly involved in the reaction, i.e., the positions of all the atoms involved in the transition state were held fixed. Heats of formation for the various transition states and the corresponding barrier heights are presented in Table 1.

**Table 1:** Heats of formation of intermediates and transition states, relative to the starting system (kcal mol^−1^)

Step	ΔH_f_	Transition state	ΔH_f_	Barrier height
1	0.00	1 → 2	+17.93	17.93
2	−2.54	2 → 3	+17.58	20.12
3	−4.83	1 → 3	+35.86	35.86
4	+4.29	4 → 5	+13.97	9.68
5	+4.60	5 → 6	+20.36	15.76
6	−5.70			

#### Validation of transition states

Transition states are characterized by the presence of precisely one imaginary normal mode of vibration representing motion in the direction connecting the reactants and products. A vibrational analysis of the five transition states produced only one imaginary mode per state, see Table 2, which confirmed that all five stationary points were indeed true transition states.

**Table 2:** Vibrational frequencies for transition state reaction coordinate normal modes

Transition state	Frequency of imaginary vibration (*i*cm^−1^)	Frequency of lowest real vibration (cm^−1^)
TS 1-2	1034.7	264.8
TS 2-3	739.7	344.6
TS 1-3	420.7	138.6
TS 4-5	1111.6	232.4
TS 5-6	826.1.3	103.5

#### Reaction without a transition state

Two reactions, 3 → 4 and 6 → 1, did not involve the making or breaking of covalent bonds. In both reactions, a product of the catalysis reaction migrated out of the active site; the amine in step 3 → 4 and the acid in step 6 → 1. Also, in step 3 → 4, a water molecule migrated into the active site. These processes involve very small energy fluctuations because all the changes that occurred were limited to the hydrogen bonding framework. To verify that no significant barriers were present, these reactions were simulated by applying a small potential that mimicked a pull in the reaction direction. As expected, all barriers encountered were very small and were identified as being associated with specific weak non-covalent interactions.

#### Properties of transition states

##### Transition state for step 1 → 2

Step 1 → 2 involves the formation of a covalent bond between the hydroxyl O_γ_ of Ser195 and the peptide carbon of Trp252. In this reaction, the hydroxyl group, originally pointing toward Ser214, rotated around the C_α_-C_β_ bond through about 180° to point toward His57. The proton then migrated away from the oxygen to form a covalent bond with N_ε2_ of His57, resulting in the formation of an imidazolium cation.

**Fig. 10:**
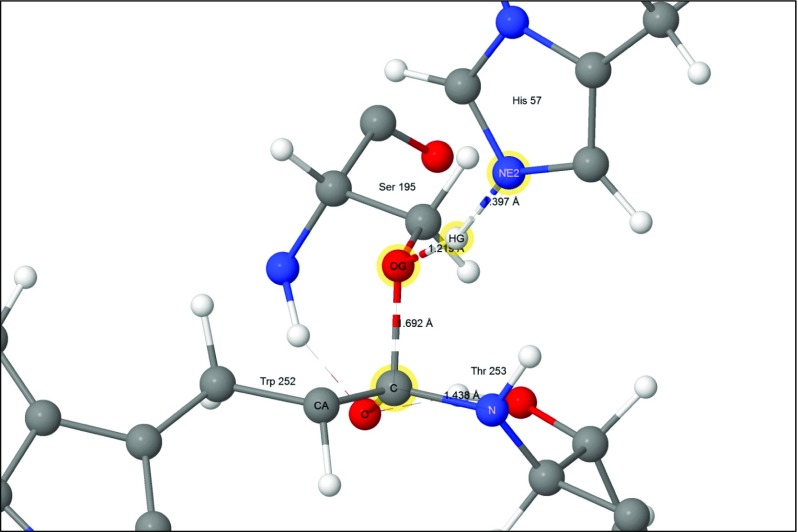
Transition state 1–2: forming the first tetrahedral intermediate and His57(+) - Asp102(−) ion-pair. Atoms involved in the reaction are indicated by halos

Four atoms were involved at the transition state: the hydroxyl oxygen atom and the hydrogen atom of Ser195, an imidazole nitrogen from His57, and the peptide carbon of Trp252, as shown in Fig. 10. The hydrogen atom formed bridging hydrogen bonds with both O_γ_ and N_ε2_, pulling the two residues together slightly. Simultaneously, the distance between residues Ser195 and Trp252 decreased as the O_γ_-C covalent bond started to form.

##### Transition state for step 2 → 3

**Fig. 11:**
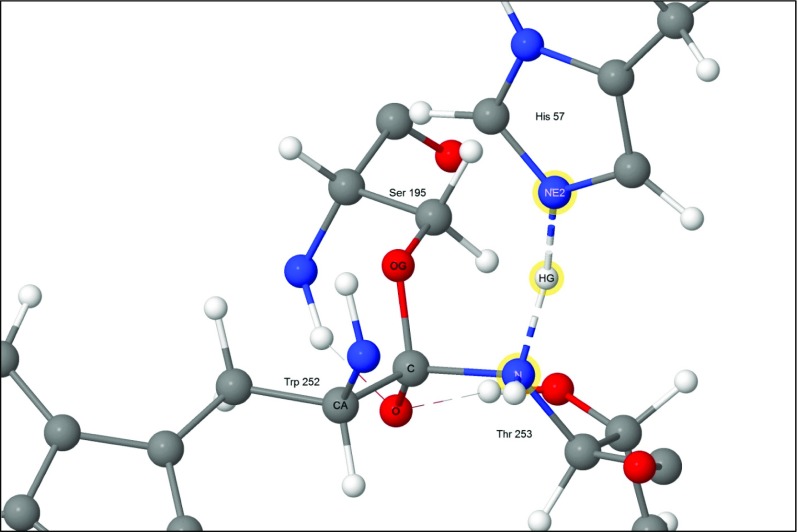
Transition state 2–3: proton migrates from His57(+) to Thr253. Atoms involved in the reaction are indicated by halos

Of the five reactions modeled, this was the simplest: the proton on N_ε2_ in the imidazolium ring of His57 migrated to the peptide nitrogen atom of Thr253, neutralizing the His57, and converting the atom Thr253 N into a quaternary ammonium cation (Fig. 11). As with step 1 → 2, at the transition state the bridging bonds pulled the two residues together slightly.

##### Transition state for the hypothetical reaction step 1 → 3

**Fig. 12:**
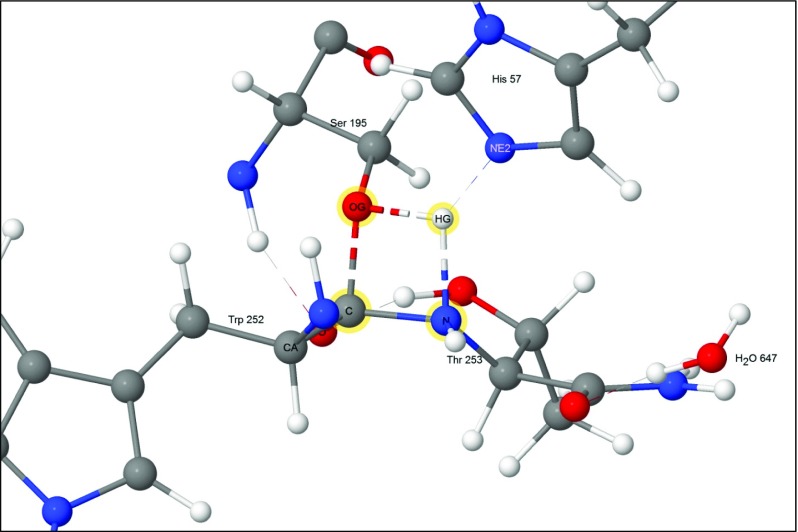
Transition state for the hypothetical reaction 1–3 where a proton migrates from Ser195 directly to Thr253. Atoms involved in the reaction are indicated by halos

Given that the presence of the two residues Asp102 and His57 reduces the height of the reaction barrier for the formation of the tetrahedral intermediate by splitting the reaction into two steps *via* step 2, the consequences of replacing the two-step process by a single step reaction would be of interest. When the transition state connecting intermediates 1 and 3 was calculated (Fig. 12), the transition state energy, as expected, was higher than that in the two-step process, by 17.93 kcal mol^−1^. Also, as expected, the transition state consisted of an almost planar quadrilateral. What was not expected was the dramatic motion of H_2_O-647, which moved several Ångstroms away from its original position (Fig. 3). This was likely a consequence of the migrating proton, Ser195 H_γ_, forming a relatively strong hydrogen bond with His57 N_ε2_, and the resulting strong steric repulsion displacing the water molecule from its original site.

##### Transition state for step 4 → 5

**Fig. 13:**
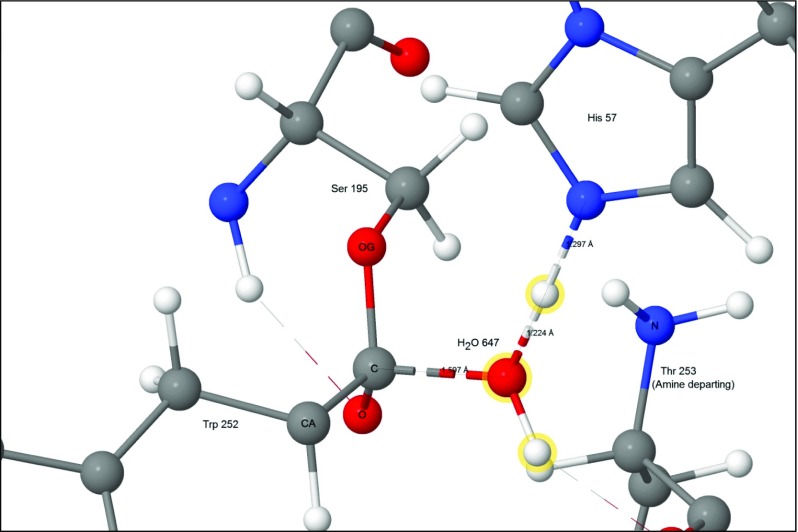
Transition state 4–5: water splitting into hydroxyl anion and a proton. Atoms of the water molecule involved in the hydrolysis are indicated by halos

The first of the two steps of the hydrolysis of the ester bond between Ser195 and Trp252 involves a water molecule splitting into a hydroxyl anion that covalently bonds to the acyl carbon on Trp252, and a proton that adds to His57 N_ε2_ (Fig. 13).

##### Transition state for step 5 → 6

**Fig. 14:**
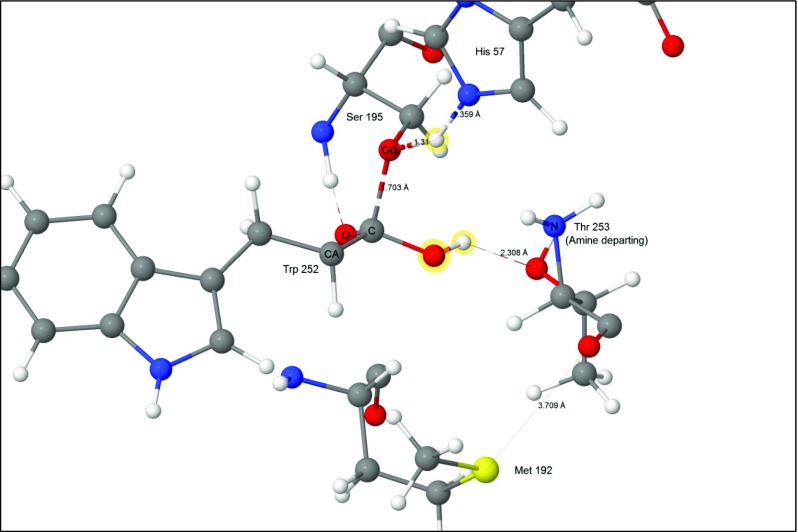
Transition state 5–6: the proton migrates from His57(+) to Ser195. Atoms of the water molecule involved in the hydrolysis are indicated by halos

In the final reaction, the proton on His57 migrated to O_γ_ on Ser195. The weak covalent bond C-O_γ_ broke during this process (Fig. 14), resulting in the formation of a carboxylic acid group on Trp252. Near the end of the reaction a salt bridge formed when the proton on the newly-formed carboxylic acid group spontaneously migrated to the terminal amine group of the departing residue Thr253.

#### Intrinsic reaction coordinate

In addition to establishing that a true transition state exists, a second test is necessary in order to confirm that the transition state corresponds with the intended reaction. This test involves displacing the coordinates in the direction of the normal mode represented by the imaginary frequency, then following the IRC until the rate of decrease in energy drops below a pre-set limit. At that point a conventional geometry optimization would then be performed. Ideally, this would result in an energy and geometry corresponding to either the intended reactant or product. A second IRC in which the atoms of the transition state were displaced in the opposite direction, i.e., reversing the direction of the normal mode, followed by geometry optimization, should then produce an energy and geometry corresponding to the intended product or reactant.

This procedure was performed using the five transition states. All ten IRC paths terminated in geometries that, by inspection using the graphical user interface JSmol [33], were between the initial transition state geometry and that of the intended stable intermediate. Unconstrained geometry optimization of the ten geometries resulted in eight continuing their motion toward the intended stable intermediate. In the other two optimizations, involving transition states 4 → 5 and 5 → 6, instead of the intended motion toward the reactants, the motion reversed and the final geometries corresponded to the products. Examination of these two unexpected results revealed that the start of each geometry optimization was normal, but this was followed by a somewhat chaotic set of optimization steps in which the heat of formation increased dramatically, corresponding to a change in motion from the downhill path to the reactants to motion on the downhill path to the products.

Further analysis revealed that, as a result of the downhill path being very shallow, the L-BFGS optimization procedure had made unexpectedly large steps, and that these large steps had resulted in the optimization moving onto the other downhill paths.

### Individual features

#### Electrostatic role of Asp102

Asp102, although an essential component of the catalytic triad, does not take an active role in any stage of the catalytic cycle. An early description [34] of its role involved a charge-relay mechanism in which the proton on Ser195 migrated to His57 N_ε2_, and the proton on His57 N_δ1_ migrated to the carboxylate of Asp102, but this was later described as being unlikely [35], and subsequent work confirmed [36] that at no point in the cycle did Asp102 exist in the protonated form. An alternative suggestion [35] was that Asp102 helped stabilize the ionized histidine in step 2, and more recently that [18] "It is a more reliable assumption that Asp102 may be involved in the stabilization of the ion-pair generated between the imidazolium ion and the negatively-charged tetrahedral intermediate, and that Asp102 may participate in the orientation of the correct tautomer of His57 relative to Ser195."

Given that the catalytic triad is an important motif that occurs frequently in hydrolytic enzymes, that the carboxylate anion is essential to the triad activity, and that two different roles have been suggested for the anion (stabilizing the His(+) and stabilizing the His(+) - tetrahedral intermediate ion pair) a computational simulation of the electrostatic properties of Asp102 in chymotrypsin was considered appropriate.

All electrostatic energies for various interactions in step 2 were evaluated using the technique described earlier. Individual components for the electrostatic energy used in Eq. 2 are given in Table 3, and the electrostatic interaction energies are shown in Table 4.

**Table 3:** Heats of formation of systems used in estimating electrostatic interactions^†^

Steps 1 and 2: Asp102-tetrahedral complex ion pair				
System	Description	Net charge	Step 1 ΔH_f_ (kcal mol^−1^)	Step 2 ΔH_f_ (kcal mol^−1^)
A	Un-modified system	−1	0.00	−2.54
B	Asp102 replaced by Ala102	0	+245.14	+253.90
C	Trp252 O replaced by F	0	+172.22	+160.84
D	Asp102 replaced by Ala102, Trp252 O replaced by F	+1	+423.29	+423.45
Step 2: Asp102-His57 ion pair				
System	Description		Net charge	ΔH_f_ (kcal mol^−1^)†
A	Un-modified system		−1	−2.54
B	Asp102 replaced by Ala102		0	+253.90
C	His57 proton on N_ε2_ deleted		−2	−69.41
D	Asp102 replaced by Ala102 and His57 proton on N_ε2_ deleted		−1	+166.38
Step 2: Asp102-His57 ion pair				
System	Description		Net charge	ΔH_f_ (kcal mol^−1^)†
A	Un-modified system		−1	−2.54
B	Proton deleted from His57		−2	−69.41
C	Trp252 O replaced by F		0	+160.84
D	Proton deleted from His57 and Trp252 O replaced by F		−1	+75.76
	Step 2 His57 - tetrahedral ion pair in mutant D102A			
System	Description		Net charge	ΔH_f_ (kcal mol^−1^)†
A	D102A mutation		0	+253.90
B	D102A mutation with proton deleted from His57		−1	+166.38
C	D102A mutation with Trp252 O replaced by F		+1	+423.45
D	D102A mutation with His57 neutral and Trp252 O replaced by F		0	+317.88

†: All heats relative to step 1 un-modified system

**Table 4:** Electrostatic interaction energies in step 2

Interaction	Electrostatic energy (kcal mol^-1^)
Asp102(−) and oxyanion site	+6.17
Asp102(−) and His57(+)	−20.65
His57(+) and oxyanion site	−18.21
His57(+) and oxyanion site in mutation D102A	−18.05

Using the results in Table 4, the degree to which the electrostatic stabilization energy of the imidazolium - tetrahedral intermediate ion pair was enhanced by the electronic effect of Asp102 was estimated to be 0.16 kcal mol^−1^. This quantity was significantly smaller than the precision of the calculations, and, for all practical purposes, could be regarded as insignificant.

##### Energy effect of the charge on Asp102 on step 2

In addition to estimating the electrostatic energies of individual interactions, it was possible to estimate the effect on the overall reaction of the negative charge on Asp102. Asp102(−) presumably works by stabilizing step 2. In reaction 1 → 2, a proton, originally on Ser195, migrated to His57 to form an imidazolium cation. This would be in close proximity to the anion Asp102, and result in the generation of a salt bridge. Using the entries for system "B" in the Asp102 - tetrahedral complex ion pair in Table 3, the change in ΔH_f_ of step 2 relative to step 1 as a result of replacing Asp102(−) by Ala102 was +8.76 kcal mol^−1^. Given that, in the unmodified system, step 2 was 2.54 kcal mol^−1^ more stable than step 1, the conclusion can be made that the D102A mutation results in a net change in the relative ΔH_f_ of step 2 of +11.30 kcal mol^−1^, and therefore the presence of Asp102(-) stabilizes step 2 by 11.30 kcal mol^-1^, relative to step 1.

This result is in agreement with the reported experimental observation [37] that "Replacement of Asp102 of trypsin with a neutral Ala residue results in a reduction of four orders of magnitude in the rate constant."

In a second simulation, the positions of all atoms in the chymotrypsin mutant D102A were optimized, resulting in the energy change due to the proton migration increasing to 13.25 kcal mol^−1^, or 15.79 kcal mol^−1^ relative to the un-mutated system. Therefore, the purely geometric effect arising from the anionic charge on Asp102 in chymotrypsin reduced the relative heat of formation of step 2 by 4.49 kcal mol^-1^. That the more important effect of D102 is electronic is “consistent with the earlier suggestion that a major function of the aspartate residue is to stabilize the transition state on the way to the ion-pair formation between the tetrahedral intermediate and the imidazolium ion” [18].

#### Hydrogen bonds in the oxyanion hole

Two properties of the oxyanion hole contribute to the catalytic ability of chymotrypsin. First, the presence of two hydrogen bond donors enhances the stabilization of the substrate in the binding pocket, and, second, during the catalytic reaction, the interaction of these two hydrogen bond plus a third hydrogen bond from Thr253 (Fig. 5) with the amide oxygen atom lowers the activation barrier so that the tetrahedral intermediate is more easily obtained. Both of these properties were reproduced in the simulations.

Partial atomic charges and hydrogen-bond distances are presented in Table 5. One of the hydrogen bonds, between Thr253 and the amide oxygen of Trp252, would not contribute to the binding of the substrate, but would contribute to the stabilization of step 2. In step 1 the partial charge on the amide oxygen of Trp252, −0.65, was small relative to the values in the next four steps, suggesting that at the start of the cycle the hydrogen bonds would be at their weakest. Also, in step 1, all three hydrogen-bond distances were large compared to their values in subsequent steps, implying that the stabilization caused by the hydrogen bonds increased as the reaction progressed. This stabilization reached a maximum at step 2, at which point the net charge on the amide oxygen was −0.95 and the three O-H hydrogen bond distances were very short (<1.7 Å) indicative [38] of strong hydrogen bonds.

**Table 5:** Partial atomic charges and distances in the oxyanion site

System	Partial atomic charges				Hydrogen bonds in the oxyanion site distance in Ångstroms between Trp252-O and:		
	Gly193-H	Ser195-H	Thr253-H_γ1_	Trp252-O	Gly193-H	Ser195-H	Thr253-H_γ1_
Step 1	+0.38	+0.34	+0.34	−0.65	2.075	3.044	2.289
TS 1-2	+0.36	+0.37	+0.35	−0.83	1.811	1.801	1.719
Step 2	+0.38	+0.40	+0.37	−0.95	1.693	1.697	1.644
TS 2-3	+0.38	+0.38	+0.37	−0.92	1.709	1.848	1.608
Step 3	+0.37	+0.37	+0.36	−0.88	1.745	1.898	1.632
Step 4	+0.37	+0.35	+0.35	−0.61	2.005	2.176	3.149

A more important property—the lowering of the activation barrier—was investigated by estimating the energies due to the hydrogen bonds. A quantitative estimate of the stabilization of step 2 due to the oxyanion hole was obtained using the method described earlier for predicting the change in energy of hydrogen bonds. This involved using the technique described earlier to selectively delete the hydrogen bonds between the substrate and residues Gly193, Ser195, and Thr253.

An estimate of the hydrogen bond stabilization energies in the oxyanion site for step 2, relative to step 1, was made using Eq. 3 and the sets of systems shown in Table 6. These, together with the electrostatic effects due to Asp102, are summarized in Table 7.

**Table 6:** Heats of formation used in estimating the change in energy of the hydrogen bonds in the oxyanion site

System	Description	ΔH_f_ (kcal mol^−1^)†
H(A)	Step 1 unmodified	0.00
H(B)	Step 2 unmodified	−2.54
X(A)	Step 1 with -NH on Gly193 replaced with -O	−32.35
X(B)	Step 2 with -NH on Gly193 replaced with -O	−21.29
X(A)	Step 1 with -NH on Ser195 replaced with -O	−26.56
X(B)	Step 2 with -NH on Ser195 replaced with -O	−15.84
X(A)	Step 1 with -OH on Thr253 replaced with -F	+6.97
X(B)	Step 2 with -OH on Thr253 replaced with -F	+11.56

†: All heats relative to step 1 un-modified system

**Table 7:** Main contributions to the stabilization of step 2

Contribution from	Energy (kcal mol^−1^)
Hydrogen bond from Gly193	−13.60
Hydrogen bond from Ser195	−13.25
Hydrogen bond from Thr253	−7.12
Hydrogen bonds from Gly193, Ser195, and Thr253	−32.06
Electronic effect of aspartate 102 anion	−11.30
Geometric effect of aspartate 102 anion	−4.49
Total effect of aspartate 102 anion	−15.79

To test whether the presence of one hydrogen bond had any effect on neighboring hydrogen bonds, the stabilization energy was evaluated when all three hydrogen bonds were considered simultaneously; this amounted to 32.06 kcal mol^−1^. When the individual contributions from each of the three hydrogen bonds were added, the stabilization energy was 33.97 kcal  mol^−1^.

## Discussion

Although locating transition states in enzyme mechanisms has been notoriously difficult, all five transition states reported here were located and refined without difficulty using the new procedure. In part, the success of this method could be attributed to the theoretically straightforward nature of enzyme-catalyzed reactions. Straightforward in the sense that the energy difference between reactants and products is normally small and that the environment of the reaction site helps orient the various parts involved in the reaction. Two computational chemistry issues, the problems in solving the SCF equations and the large size of the systems involved, have to a large degree, been solved by the use of the MOZYME technique; and one issue, the considerable number of computationally-demanding SCF calculations needed in locating transition states, was addressed by only performing a SCF calculation when the bias in Eq. 1 was changed.

### Barrier heights

An estimate of the accuracy of barrier heights, Table 1, can be obtained from a survey [39] which reported that, for four enzymes that did not contain metal atoms, the mean absolute difference for barrier heights calculated using PM7 compared to the reference method B3LYP/6-311+G(2d,2p) [LANL2DZ]//B3LYP/6-31G(d,p) was 7 kcal mol^-1^. An unfortunate consequence of the use of a restricted basis set in semiempirical methods is the reduction in the accuracy of prediction of transition state barrier heights. Fortunately, geometries are less affected, so one way to improve the accuracy of prediction of barrier heights would be to use the semiempirical transition state geometry as a starting point for a more accurate calculation, thus avoiding the computationally-intensive search for transition state stationary points.

Although absolute errors in barrier heights predicted using PM7 are large, errors in differences in barrier heights are likely to be smaller due to cancellation of errors, and therefore some limited inferences could be made. Thus, as expected, the barrier height for the un-catalyzed reaction 1 → 3 was significantly higher than those in the two-step process 1 → 2 → 3, and the lowest barrier was for the ionization of the water molecule in step 4 → 5.

### Role of methionine 192

Despite the fact that the sulfur atom in residue Met192 is far from any of the atoms in the active site (Fig. 8), it did move significantly in all of the reactions modeled, reaching a maximum of almost 2 Å in the reaction 5 → 6 (Fig. 14). Residue Met192 is very flexible and lies on the surface of chymotrypsin, however the magnitude of the excursion of its sulfur atom in reaction 5 → 6 was unexpectedly large.

An estimate of the influence of the sulfur atom in Met192 on the reaction was obtained by replacing the CH_3_-S group in Met192 with a hydrogen atom, so that the side-chain became -CH_2_-CH_3_, followed by re-evaluating the energies of the steps in the catalytic cycle. Earlier tests [13] using simulated mutations had shown that only residues involved in docking affect relative energies in the docking site, and presumably a similar situation would apply to reaction sites. The results presented in Table 8 show that the presence of Met192 has a significant influence on the various energies, and therefore Met192 is predicted to be involved in the catalysis.

**Table 8:** Heats of formation for intermediates and transition states, where the side-chain of Met192 was replaced with -CH_2_CH_3_ (kcal mol^−1^)

Step	ΔH_f_^†^	Change^‡^	Transition state	ΔH_f_^†^	Change^‡^	Barrier Height	Change^‡^
1	0.00		1 → 2	+18.42	+0.49	18.42	+0.49
2	+2.48	+5.02	2 → 3	+20.35	+2.77	17.87	−2.25
3	−1.19	+3.64	1 → 3	+39.40	+3.54	39.40	+3.54
4	+5.59	+1.30	4 → 5	+14.39	+0.41	8.79	−0.89
5	+1.59	−3.01	5 → 6	+24.35	+3.99	22.76	+7.00
6	−2.51	+3.19					

† ΔH_f_ relative to that of step 1.

‡Changes are relative to the un-mutated system

With the exception of step 5, the effect of the mutation was to increase the relative energies of the intermediates, and, more importantly, that of all the transition states. From this, it follows that the presence of the methionine side-chain in native chymotrypsin is predicted to help stabilize all the transition states and therefore contribute to the rate of catalysis.

An examination of the environment of the sulfur atom in Met192 failed to provide a convincing explanation for its effect on the heats of formation of the transition state. Reaction 5 → 6 was the transition state with the highest energy, at 20.36 kcal mol^−1^, in agreement with an earlier report [31], and would therefore be the rate-determining step, and thus the most important system to be influenced by the sulfur atom. In this reaction the presence of the sulfur atom contributed a stabilizing energy of 3.99 kcal mol^−1^. Examination of this system showed that, at the start of this reaction (Fig. 8), the imidazolium hydrogen attached to N_ε2_ formed a very strong hydrogen bond with the hydroxyl oxygen on the tetrahedral carbon anionic site on Trp252. This oriented the hydroxyl hydrogen to point toward the peptide oxygen atom of Thr253, forming a normal hydrogen bond. In turn, that oxygen atom was 3.27 Å from the sulfur. During the reaction, the imidazolium ring rotated so that the migrating proton formed a hydrogen bond with O_γ_ on Ser195, which effectively destroyed the hydrogen bond between the hydroxyl oxygen on Trp252 and N_ε2_ on His57, and allowed the hydroxyl hydrogen to rotate to form a hydrogen bond with the hydroxyl oxygen of the departing Thr253 (Fig. 14). Following this change in hydrogen bonding, the peptide oxygen-sulfur distance increased to 3.71 Å. Presumably these changes affect the heat of formation of the transition state, but despite the phenomena in reaction 5 → 6 being easy to observe, the cause of the change in heat of formation still remains obscure. Nevertheless, the fact remains that the presence of Met192 is predicted to lower the activation barrier, and thus enhance the catalytic power of the enzyme, and this prediction is supported by experimental results [40–42] that show that the presence of Met192 has an observable effect on catalytic activity.

### Flexibility in the active site

A superficial examination of the environment of the catalytic triad, the substrate, and the oxyanion hole, might give the impression that these moieties have considerable flexibility, but a more detailed examination would reveal that almost all motion was constrained by the hydrogen bond network, with both the substrate and its environment bound together like “Gulliver: a giant, constrained by a multitude of weak bonds” [43]. This is dramatically illustrated by the carboxylate functional group in the first residue in the catalytic triad, Asp102. This group forms one simple, very strong, hydrogen bond with His57 H_δ1_, and also forms hydrogen bonds with Ala56 H, His57 H, and Ser214 H_γ_, the overall result being that the Asp102 carboxylate group is held rigidly in place by the surrounding protein. Similarly, His57 N_ε2_ forms three hydrogen bonds, with Thr253 H, Leu254 H, and with a hydrogen atom in H_2_O-647. In the conventional hydrogen bonding picture of chymotrypsin, His57 N_ε2_ also forms a hydrogen bond with Ser195 H_γ_, but rather than His57 N_ε2_ participating in four hydrogen bonds, PM7 predicts that the hydrogen bond to Ser195 is missing, and instead, Ser195 H_γ_ hydrogen-bonds with Ser214 O. A similar analysis of the oxyanion hole reveals that, within the hole itself, residues Gly193 and Ser195 each form only one intra-protein hydrogen bond and each form one hydrogen bond with a water molecule, and therefore these two residues exhibit somewhat enhanced flexibility. Conversely, the carboxylate group on the middle residue, Asp194, forms three strong inter-protein hydrogen bonds, and is therefore much less flexible.

### Fission of the peptide bond

The process of breaking the peptide bond occurs in the first three steps of the mechanism. At the start of the reaction cycle, step 1, the properties of the Trp252-Thr253 peptide bond have the expected values for a normal peptide bond (Table 9). As the reaction proceeds, the C-N bond-length increases monotonically, accompanied by a simultaneous monotonic decrease in the bond order. An examination of the covalent interactions present at each stage of the reaction provides an explanation for this sequence.

**Table 9:** Bond orders, charges, and distances in the peptide bond between Trp252 and Thr253

Stationary points	Valencies					Bond orders				Partial atomic charges					Bond lengths (Å)	
	O_γ_^1^	C	N	O	H_γ_^2^	C-O_γ_	C-N	C=O	N-H_γ_	O_γ_	C	N	O	H_γ_	C-N	C=O
Step 1	1.99	3.80	3.20	1.95	0.86	0.00	1.18	1.57	0.00	−0.62	+0.62	−0.57	−0.65	+0.37	1.370	1.226
TS 1-2	2.10	3.72	3.23	1.73	0.80	0.43	0.94	1.32	0.00	−0.61	+0.74	−0.67	−0.83	+0.45	1.438	1.259
Step 2	2.10	3.77	3.19	1.56	0.86	0.81	0.84	1.11	0.00	−0.56	+0.71	−0.68	−0.95	+0.38	1.502	1.300
TS 2-3	2.14	3.78	3.20	1.62	0.78	0.86	0.75	1.16	0.39	−0.50	+0.69	−0.59	−0.92	+0.47	1.560	1.284
Step 3	2.17	3.76	3.41	1.69	0.89	0.91	0.60	1.23	0.84	−0.46	+0.68	−0.39	−0.88	+0.34	1.660	1.268
Step 4	2.33	3.75	3.00	2.02	0.92	1.08	0.00	1.66	0.89	−0.36	+0.60	−0.67	−0.61	+0.29	3.67^3^	1.211

1: oxygen on Ser195. 2: atom originally on Ser195. 3: covalent bond broken.

Throughout the reaction the valence [44] of the carbon atom varies between 3.72 and 3.80, close to the expected formal valency of 4. Initially, it forms conventional single bonds with the adjacent atoms C_α_ and N, and a partial double bond with O. At the first transition state, TS 1-2, a new, partial, covalent bond exists between it and O_γ_ on Ser195. This bond would be only partial because O_γ_ still forms a partial covalent bond with H_γ_ on Ser195, with the H_γ_ forming a new partial bond with N_ε2_ on His57. As a direct consequence of this, at TS 1–2, the order of the other bonds to the carbon, specifically the C=O and C-N bonds, would be reduced. With the completion of the first reaction, step 2, a normal covalent single bond connects Trp252 C and Ser195 O. This increase in the covalent bond character on carbon then contributes to a further weakening of the C=O and C-N bond orders. A similar sequence of events occurs at points TS 2–3 and step 3, where the H_γ_ atom from Ser195 that had migrated to His57 N_ε2_ continues its migration to Thr253 N; at the transition state, Thr253 N forms a partial covalent bond with the migrating hydrogen atom, and at the next stationary point, step 3, its bond order is increased to that of a single covalent bond. The motions from step 2 to TS 2–3 and from TS 2–3 to step 3 both result in a decrease in the order of the C-N bond, and a concomitant increase in the C=O bond order.

Hypothetically, if the changes in the C-N bond order were to be ignored, then Lewis theory would predict that at step 2 the carbonyl oxygen atom would acquire a formal negative charge, and at step 3 the nitrogen atom would acquire a formal positive charge. To some degree this is reflected in the partial atomic charges on these two atoms. In step 2, the partial charge on the oxygen atom is at its maximum negative, at −0.95, and in step 3 the partial charge on nitrogen is at its minimum negative, at -0.39.

The structure in step 3 is unusual in that the presence of a partial covalent C-N bond confers some zwitterionic character to the peptide group. Both the zwitterionic character and the partial covalent character could be eliminated without making or breaking any other covalent bonds by the simple action of increasing the C-N bond-length, i.e., by an activationless reaction. This process was simulated, and during the formation of the expected ester and amine, small fluctuations, mainly increases, in energy occurred. These were most likely caused by the loss of hydrogen bonds.

### Shape of reaction path

Each half of a reaction path can be represented by a curved line on the mass-weighted Cartesian PES connecting a transition state to a stable minimum by following the steepest gradient at each point. A complete reaction path for any specific reaction would obviously involve two half-reaction paths: the path from one intermediate to the transition state followed by the path from the transition state to the other intermediate. Reaction paths were investigated using two methods with the more detailed method involving the explicit mapping of the IRC. Each point evaluated was represented by three measures: the energy of the system at that point, the geometry, and the distance from the transition state geometry. This latter quantity was used as the definition of the position on the reaction coordinate. Individual features of interest within each reaction were then examined in detail using JSmol. In particular, each reaction in the catalytic cycle was checked to verify that both termini of the IRC corresponded with the expected intermediates.

Another method, less informative but considerably easier to evaluate, involved calculating the angle on the Cartesian PES between the reactants, the transition state, and the products. In the case of a very simple idealized reaction where an atom, originally bonded to one atom, migrated in a straight line to bond to another atom, and all other atomic motions were negligible, the angle would be 180°. Angles significantly smaller than 180° would then indicate a curved reaction path.

For the reactions 1 → 2 and 2 → 3 the distances between pairs of points and the resulting angles are presented in Table 10. Less well-defined but nonetheless interesting quantities involve the relationship of the transition state to points near the bottom of the reaction barrier, points **B** and **F** in Fig. 1. These quantities are also presented in Table 10.

**Table 10:** Distances and angles for transition states

Systems			From stationary points (points **A** and **G** in Fig. 1)		From bottom of reaction barrier (points **B** and **F** in Fig. 1)	
Label	A	B	Distance A-B (Å)	Angle a-b (°)	Distance A-B (Å)	Angle a-b (°)
a	Step 1	TS 1-2	127.39		9.06	
	Step 1	Step 2	249.79	129.99	8.80	59.22
b	TS 1-2	Step 2	148.06		8.75	
a	Step 2	TS 2-3	115.13		8.32	
	Step 2	Step 3	224.21	100.42	7.17	54.17
b	TS 2-3	Step 3	172.70		7.30	

For these reactions, the results in Table 10 show that all the distances between stationary points and their transition states were over 100 Å and the contained angles at the transition states were more than 100°. However, these apparently reasonable quantities were a consequence of the almost flat PES between the base of the reaction barrier and the corresponding stationary point.

Theoretically, a more informative measure of the shape of the barrier could be obtained by using the geometries of a transition state and the two points on the reaction coordinate where the slope of the barrier became small, i.e., the bottom of the barrier. This effectively excludes the essentially flat plain between the base of the barrier and the precise stationary point representing the intermediates. For reaction 1 → 2, this gave a barrier width (Table 10) of ca. 18 Å and ca. 16 Å for reaction 2 → 3.

What was unexpected were the contained angles: for both reactions, these were less than 60°. Given that at the transition state all vibrational modes, including the reaction normal coordinate, would be parabolic in energy and symmetric in displacement, i.e., all contained angles would be exactly 180°, and that the angles between the stationary points and the transition state were over 100°, the small angles between the base and top of the barrier warranted further investigation.

An explanation, applicable to both reactions, was provided by a careful examination of the geometries on the reaction coordinate; this can best be understood by reference to reaction 2 → 3, in which the entire reaction consists of the migrating proton moving from His57 to the peptide nitrogen between Trp252 and Thr253.

At the transition state, the migrating proton was approximately midway between the donor and acceptor atoms, and formed a bridging covalent bond with both. This had the effect of reducing the interatomic distances between the atoms of the donor and acceptor groups from their values in the intermediates. The largest intensity in the reaction coordinate vibration normal mode was on the hydrogen atom, indicating that the initial motion was dominated by migration of the proton, and at that point, the contained angle was, by definition, 180°.

With increasing distance from the transition state, one or other of the bridging covalent bonds rapidly weakened. This resulted in an increase in the donor-acceptor separation as the system relaxed, but because the increase in separation was essentially symmetric, the increase in distance between the geometries of the reactants and products was disproportionately small compared to the distances between the reactants and products and the transition state. This effect, and not any curvature of the reaction path, was responsible for the observed small contained angle.

At still greater reaction coordinate distances, the geometric effects resulting from the descent of the barrier, effects initially localized to the region of the reactive site, slowly propagated throughout the entire system, ultimately dying out when the stationary points were reached. In this region of the PES all individual atomic motions were small, in the order of hundredths of an Ångstrom, but their overall effect was large, amounting to over ten times the total motion of the atoms in the reactive site.

Of the two methods for representing the reaction coordinate, the IRC was by far the more useful in that it gave a detailed and informative description of the reaction path. The use of contained angles, although superficially appealing because it would appear to discriminate between simple and more complicated reaction paths, must be deprecated on the grounds that when it was applied to even a very simple reaction, here the migration of a proton from His57 to Thr253, the results were not only not useful or probative, they were positively misleading.

### Summary of the catalytic cycle

Using only the results reported here, the mechanism employed by chymotrypsin in the proteolytic hydrolysis of the substrate Gly250-Ala251-Trp252-Thr253-NH_2_ can be summarized as follows:

Step 1 At the start of the catalytic cycle the substrate was docked in the binding site and held in place by weak non-covalent bonds. Within the reaction site, the oxyanion hole formed two normal hydrogen bonds with the peptide oxygen on Trp252. Trp252 also formed an intra-substrate hydrogen bond with the adjacent residue, Thr253. On the other side of the substrate, His57 was oriented so that it formed a very strong hydrogen bond with the only ionized residue in the active site, Asp102(−).
Step 2 The catalytic cycle began with the hydroxyl oxygen on Ser195 forming a covalent bond with the peptide carbon of Trp252, resulting in the formation of a tetrahedral intermediate. Several other changes occurred simultaneously:
  Charge separation occurs as the hydroxyl hydrogen migrates from Ser195 to His57 to form a histidinium cation. This ionization results in the formation of a salt bridge between Asp102 and His57, as can be seen by the changes in partial atomic charges on various atoms in the active site (Table 11) and on fractional charges on residues (Table 12). The net change in charge at the His57 site was +0.972, close to the expected change of +1.000.

**Table 11:** Partial atomic charges in intermediates 1 and 2

Atom		Step 1	Step 2	Δ
Cationic site				
His57	C_β_	−0.31	−0.30	+0.01
His57	1H_β_	+0.20	+0.21	+0.01
His57	2H_β_	+0.21	+0.22	+0.01
His57	C_γ_	+0.00	+0.03	+0.03
His57	N_δ1_	−0.35	−0.33	+0.02
His57	C_δ2_	−0.10	−0.06	+0.04
His57	C_ε1_	+0.08	+0.16	+0.08
His57	N_ε2_	−0.50	−0.25	+0.25
His57	H_δ1_	+0.38	+0.41	+0.03
His57	H_δ2_	+0.20	+0.24	+0.04
His57	H_ε1_	+0.20	+0.24	+0.04
His57	H_γ2_	-	+0.38	+0.38
Oxyanion hole				
Ser195	C_β_	+0.00	−0.02	−0.02
Ser195	O_γ_	−0.62	−0.56	+0.06
Ser195	H_γ_	+0.37	-	−0.37
Trp252	C_α_	+0.03	+0.04	+0.01
Trp252	N	−0.57	−0.59	−0.02
Trp252	C	+0.62	+0.71	+0.09
Trp252	O	−0.65	−0.95	−0.30
Thr253	N	−0.57	−0.68	−0.11
Thr253	H	+0.35	+0.29	−0.06
Thr253	C_α_	−0.08	−0.08	+0.00

**Table 12:** Fractional charges on residues in intermediates 1 and 2

Residue	Step 1	Step 2	Δ
Cationic site			
Gly43	+0.067	+0.089	+0.022
His57	+0.059	+0.947	+0.888
Asp102	−0.816	−0.789	+0.027
Ser214	−0.095	−0.060	+0.035
Oxyanion (anionic) site			
Met192	−0.007	−0.049	−0.042
Asp194	−0.922	−0.960	−0.038
Ser195	+0.018	−0.267	−0.285
Gly196	−0.009	−0.039	−0.030
Ile212	−0.014	−0.024	−0.010
Trp215	+0.098	+0.041	−0.057
Trp252	+0.045	−0.233	−0.278
Thr253	+0.000	−0.281	−0.281
Leu254	+0.003	−0.011	−0.014
  Electrostatic interaction between the newly-formed His57(+) and Asp102(−) stabilized step 2 by 20.65 kcal mol^−1^.
  The loss of a unit positive charge to His57 resulted in the formation of a unit negative charge at the site of the newly-formed covalent bond between Ser195 and Trp252. The predicted net change at the oxyanion site was −1.035, close to the expected -1.000. Within the oxyanion site, the largest change in atomic charge occurred at the peptide oxygen, which increased from −0.65 to -0.95, as a result of the formation of the anionic tetrahedral intermediate. This, in turn, caused the three hydrogen bonds in the oxyanion site to shorten and their energy to increase by 32.06 kcal mol^−1^.
  The electrostatic interaction energy of the negative charge in the oxyanion site with the positive His57 stabilized the system by 18.21 kcal mol^−1^. This stabilization was essentially unaffected by the presence of the ionized Asp102, in variance with an earlier conjecture [35] that the presence of ionized Asp102 would enhance the stability of the His57-oxyanion site interaction.
  The peptide bond length increased by 0.13 Å, indicating a slight weakening of that bond.
  Migration of the proton from His57(+) to the peptide nitrogen on Thr253 resulted in the formation of a zwitterionic bond. This step caused a further increase in the length of the peptide bond.
  The weakened peptide bond broke in an activationless reaction. One fragment, the amine H_2_N-CH(CH(CH_3_)OH)-CONH_2_, moved several Ångstroms away from its original position. The other fragment, consisting of the tripeptide Gly250-Ala251-Trp252, was still covalently bound to the enzyme by an acyl group between Trp252 and Ser195.
  Water molecule H_2_O-647 split to form a hydroxide group and a proton. The hydroxide formed a covalent bond to the acyl carbon of Thr252, converting it into a second tetrahedral anion intermediate. Similar to the first such intermediate, this one also formed hydrogen bonds *via* its keto oxygen atom with atoms in the oxyanion hole. Also, as in step 2, the proton migrated to His57 where the resulting ionized residue was once again stabilized by electrostatic interaction with Asp102(−).
  The proton on His57 migrated by way of a bridging hydrogen bond to O_γ_ on Ser195, causing the bond between the O_γ_ and the acyl carbon to break, and resulting in the re-generation of the unbound triad residue Ser195, and the formation of a carboxylic acid group on Trp252. This was followed immediately by the activationless migration of the carboxylic acid proton to the amine group on Thr253 resulting in the formation of a salt bridge.

At the end of the cycle, the products of hydrolysis would still be in close proximity to the active site. Their departure was not modeled given that, in vivo, these would naturally diffuse away from the active site leaving it ready to accept a new substrate.

## Conclusions

Several features of the chymotrypsin mechanism were explored. Of these, the most important was the reaction cycle itself. All six stable intermediates and transition states for all steps that involved activation barriers were reproduced, and the resulting geometries were in good agreement with those expected. A postulated intermediate that had a shallow minimum, between steps 5 and 6, was not reproduced. Transition states were validated by vibrational frequency analysis, and confirmation that each transition state connected the expected intermediates was obtained by intrinsic reaction coordinate analysis.

Several features related to the catalytic cycle were explored. A hypothetical step that involved the one-step addition of the serine hydroxyl group across the peptide bond being hydrolyzed was modeled. As expected, the activation barrier was significantly higher than in the standard two-step process in which the proton first migrates onto the histidine then migrates to the peptide nitrogen. Other hypothetical reactions, not reported here, were modeled, and in every case the barriers were higher than in the accepted mechanism.

Two important energy terms frequently used in describing individual features of reaction steps were isolated:

- The electrostatic energy between various charged sites, e.g., between the histidinium cation and the aspartate anion in the catalytic triad. All essential similar features within the mechanism were successfully described.
- The role of the hydrogen bonds in the oxyanion hole in stabilizing various intermediates. Although these bonds exist at all stages of the peptide bond fission, in their absence the energy of some intermediates would be considerably higher, providing strong evidence of their importance in facilitating the reaction. An analysis of the changing atomic partial charges and interatomic distances in the fission reaction showed that the simulation was in complete agreement with the expected mechanism.

Residue Met192 was predicted to move significantly in all steps, with the maximum motion, almost 2 Å, occurring in the 5 → 6 reaction. The presence of Met192 helped reduce the energy of the transition state, but although simple hydrogen-bonding structures were found in both the starting point for that reaction and in the transition state, no persuasive explanation was found for the stabilizing effect of Met192 on the transition state.

In only one detail was there a disagreement between the predicted results and previously-published results. Earlier descriptions of the role of the buried aspartate suggested that its purpose was to help stabilize the histidinium-oxyanion site electrostatic interaction. However, when this interaction was modeled, the contribution due to the aspartate was found to be insignificant. Instead, the simulations predicted that the role of the buried aspartate was to stabilize the reaction intermediate histidinium cation by forming a salt bridge.

The chymotrypsin mechanism was selected for study because it has been well-documented and because it represents a typical closed-shell enzyme-catalyzed reaction. Computationally, all stages of the study were uncomplicated. Once appropriate starting geometries were available for the various intermediates, conventional unconstrained global optimizations were performed without difficulty. All transition states were located using a new transition state location method and the geometries of appropriate pairs of intermediates. Validation of transition states was done using established methods. Using a graphical user interface, mutation of individual systems for use in quantifying energy terms, such as electrostatic interactions between ionized sites and the stabilizing effect of hydrogen bonds, was also straightforward. The most demanding stage was the initial examination of the mechanism to determine the sequence and order of the computational experiments.

Vast amounts of data were generated during the current investigation. Initially, only those data of immediate relevance to the catalytic cycle were abstracted, but as the investigation proceeded, many new questions arose. In each case, a re-examination of the data provided information that allowed the question to be answered. In these analyses the JSmol graphical user interface was invaluable. The fact that answers to all the questions that arose were already present in the results and only needed to be extracted indicated a high degree of internal consistency in the various models, and provided confidence in the overall validity of the simulations.

Without modification, the techniques used here could be applied to other systems. In this work, a readily-available computational method was used to produce a highly-detailed description of the mechanism of the catalytic cycle in an enzyme. In all important details the description of the cycle and the conclusions resulting from this work agreed with those obtained from experimental work. In addition to modeling likely mechanisms, less likely and hypothetical mechanisms can also be modeled. An example reported here involved modeling a hypothetical reaction step in which a proton migrated from Ser195, one of the residues in the catalytic triad, directly to the nitrogen of the amide bond that was to be hydrolyzed. This step did not involve the other two residues in the triad, and therefore would be unlikely to occur in vivo. As expected, the computational results indicated that this reaction was energetically unfavorable and therefore unlikely to occur in nature.

Given that the only experimental data used in this investigation was an X-ray structure, the methods used here would also be suitable for the de novo prediction of catalytic mechanisms in enzymes when experimental data were either not available or inconclusive. When mechanisms are not available, a hypothetical mechanism could be constructed, the validity of which could then be tested using computational modeling. The results would either support the candidate mechanism, or indicate which steps were unrealistic. In the latter case, modifications could be made to the mechanism and the simulation re-run. Of course, as new experimental data become available, the predicted mechanism would either be confirmed or modified as necessary.

## References

[1] Stewart JJP (2013) Optimization of parameters for semiempirical methods VI: more modifications to the NDDO approximations and re-optimization of parameters. J Mol Modeling 19:1–32 10.1007/s00894-012-1667-x PMC3536963 23187683

[2] Grimme S, Antony J, Ehrlich S, Krieg H (2010). A consistent and accurate ab initio parametrization of density functional dispersion correction (DFT-D) for the 94 elements H-Pu. J Chem Phys.

[3] Grimme S (2012) Supramolecular binding thermodynamics by dispersion-corrected density functional theory. Chem Eur J 9955:9964 10.1002/chem.201200497 22782805

[4] Risthaus T, Grimme S (2013) Benchmarking of London dispersion-accounting density functional theory methods on very large molecular complexes. J Chem Theory Comput 9:1588–1591 10.1021/ct301081n 26587619

[5] Korth M (2010) Third-generation hydrogen-bonding corrections for semiempirical QM methods and force fields. J Chem Theory Comput 6:3808–3816

[6] Korth M, Pitonák M, Rezác J, Hobza P (2010) A transferable H-bonding correction for semiempirical quantum-chemical methods. J Chem Theory Computation 6:344–352 10.1021/ct900541n 26614342

[7] Dobeš P, Fanfrlík J, Rezác J, Otyepka M, Hobza P (2011). Transferable scoring function based on semiempirical quantum mechanical PM6-DH2 method: CDK2 with 15 structurally diverse inhibitors. J Comput Aided Mol Des.

[8] Rezac J, Hobza P (2011). A halogen-bonding correction for the semiempirical PM6 method. Chem Phys Lett.

[9] Rezac J, Hobza P (2012) Advanced corrections of hydrogen bonding and dispersion for semiempirical quantum mechanical methods. J Chem Theory and Comp 8:141–151 10.1021/ct200751e 26592877

[10] Brandon CJ, Martin BP, McGee KJ, Stewart JJP, Braun-Sand SB (2015). An approach to creating a more realistic working model from a protein data bank entry. J Mol Modeling.

[11] Berman HM, Westbrook J, Feng Z, Gilliland G, Bhat TN, Weissig H, Shindyalov IN, Bourne PE (2000) The Protein Data Bank. Accessed http://www.pdb.org 10.1093/nar/28.1.235 PMC102472 10592235

[12] Martin BP, Brandon CJ, Stewart JJ, Braun‐Sand SB (2015). Accuracy issues involved in modeling in vivo protein structures using PM7. Proteins: Struct, Funct, Bioinforma.

[13] Stewart JJ (2016). A method for predicting individual residue contributions to enzyme specificity and binding-site energies, and its application to MTH1. J Mol Model.

[14] Ryan H, Carter M, Stenmark P, Stewart JJP, Braun-sand SB (2016) A comparison of X-ray and calculated structures of the enzyme MTH1. J Mol Modeling doi:10.1007/s00894-016-3025-x 10.1007/s00894-016-3025-x PMC4923096 27350386

[15] Stewart JJP (2007) Optimization of parameters for semiempirical methods V: modification of NDDO approximations and application to 70 elements. J Mol Modeling 1173 10.1007/s00894-007-0233-4 PMC2039871 17828561

[16] Hedstrom L, Szilagyi L, Rutter WJ (1992) Converting trypsin to chymotryspin: the role of surface loops. Science 255(5049):1249 10.1126/science.1546324 1546324

[17] Perona JJ, Hedstrom L, Rutter WJ, Fletterick RJ (1995). Structural origins of substrate discrimination in trypsin and chymotrypsin. Biochemistry.

[18] Polgar L (2005). The catalytic triad of serine peptidases. Cell Mol Life Sci CMLS.

[19] Klamt A, Schüürmann G (1993) COSMO: a new approach to dielectric screening in solvents with explicit expressions for the screening energy and its gradient. J Chem Soc Perkin Trans 2:799–805

[20] Stewart JJP (1989) Example of the advantage of the BFGS geometry optimizer over the DFP method. Comput Chem 13(2):157–158

[21] Nocedal J (1980) Updating quasi-Newton matrices with limited storage. Math Comput 35:773–782

[22] Liu DC, Nocedal J (1989) On the limited memory method for large scale optimization. Mathematical Programming B 45:503–528

[23] Govind N, Petersen M, Fitzgerald G, King-Smith D, Andzelm J (2003). A generalized synchronous transit method for transition state location. Comput Mater Sci.

[24] Lever G, Cole DJ, Lonsdale R, Ranaghan KE, Wales DJ, Mulholland AJ, Skylaris C-K, Payne MC (2014) Large-scale density functional theory transition state searching in enzymes. J Phys Chem Lett 5(21):3614–3619 10.1021/jz5018703 26278727

[25] Zhang X-J, Shang C, Liu Z-P (2013). Double-ended surface walking method for pathway building and transition state location of complex reactions. J Chem Theory Comput.

[26] Maeda S, Harabuchi Y, Ono Y, Taketsugu T, Morokuma K (2015) Intrinsic reaction coordinate: calculation, bifurcation, and automated search. Int J Quantum Chem 115(5):258–269

[27] Stewart JJP (2008) Application of the PM6 method to modeling proteins. J Mol Model 15:765–805 10.1007/s00894-008-0420-y 19066990

[28] Behn A, Zimmerman PM, Bell AT, Head-Gordon M (2011) Incorporating linear synchronous transit interpolation into the growing string method: algorithm and applications. J Chem Theory Comput 7(12):4019–4025 10.1021/ct200654u 26598348

[29] Baker J (1986) An algorithm for the location of transition states. J Comp Chem 7:385

[30] Fukui K (1981) The path of chemical reactions—the IRC approach. Accounts Chem Res 14:363–368

[31] Topf M, Richards WG (2004). Theoretical studies on the deacylation step of serine protease catalysis in the gas phase, in solution, and in elastase. J Am Chem Soc.

[32] Stewart JJP (2016) MOPAC2016. Stewart Computational Chemistry, Colorado Springs

[33] Hanson RM, Prilusky J, Renjian Z, Nakane T, Sussman JL (2013) JSmol and the next-generation web-based representation of 3D molecular structure as applied to proteopedia. Israel J Chem 53(3–4):207–216. doi:10.1002/ijch.201300024

[34] Blow DM, Birktoft J, Hartley BS (1969). Role of a buried acid group in the mechanism of action of chymotrypsin. Nature.

[35] Polgár L, Bender ML (1969). The nature of general base-general acid catalysis in serine proteases. Proc Natl Acad Sci.

[36] Kossiakoff AA, Spencer SA (1981). Direct determination of the protonation states of aspartic acid-102 and histidine-57 in the tetrahedral intermediate of the serine proteases: neutron structure of trypsin. Biochemistry.

[37] Náray-Szabó G, Oláh J, Krámos B (2013). Quantum mechanical modeling: a tool for the understanding of enzyme reactions. Biomolecules.

[38] Rajagopal S (1832). Vishveshwara S (2005) Short hydrogen bonds in proteins. Fed Eur Biochem Soc (FEBS) J.

[39] Kromann JC, Christensen AS, Cui Q, Jensen JH (2016). Towards a barrier height benchmark set for biologically relevant systems. PeerJ.

[40] Lawson WB, Schramm H-J (1965) Modification of a methionine residue near the active site of chymotrypsin by p-nitrophenyl bromoacetyl-α-aminoisobutyrate. Biochemistry 4(3):377–386 10.1021/bi00879a001 14311607

[41] Taylor RP, Vatz JB, Lumry R (1973). Control of conformation of α-chymotrypsin through chemical modification. Biochemistry.

[42] Gertler A, Walsh KA, Neurath H (1974). Catalysis by chymotrypsinogen: increased reactivity due to oxidation of methionine 192. FEBS Lett.

[43] Jeffrey GA, Jeffrey GA (1997). An introduction to hydrogen bonding.

[44] Armstrong DR, Perkins PG, Stewart JJP (1973). Bond Indices and Valency.

